# Supplementary figures and images for: Regulation of H9C2 cell hypertrophy by 14-3-3η via inhibiting glycolysis
Source: PLoS One. 2024 Jul 22;19(7):e0307696. doi: 10.1371/journal.pone.0307696 (PMC11262655; doi:10.1371/journal.pone.0307696)

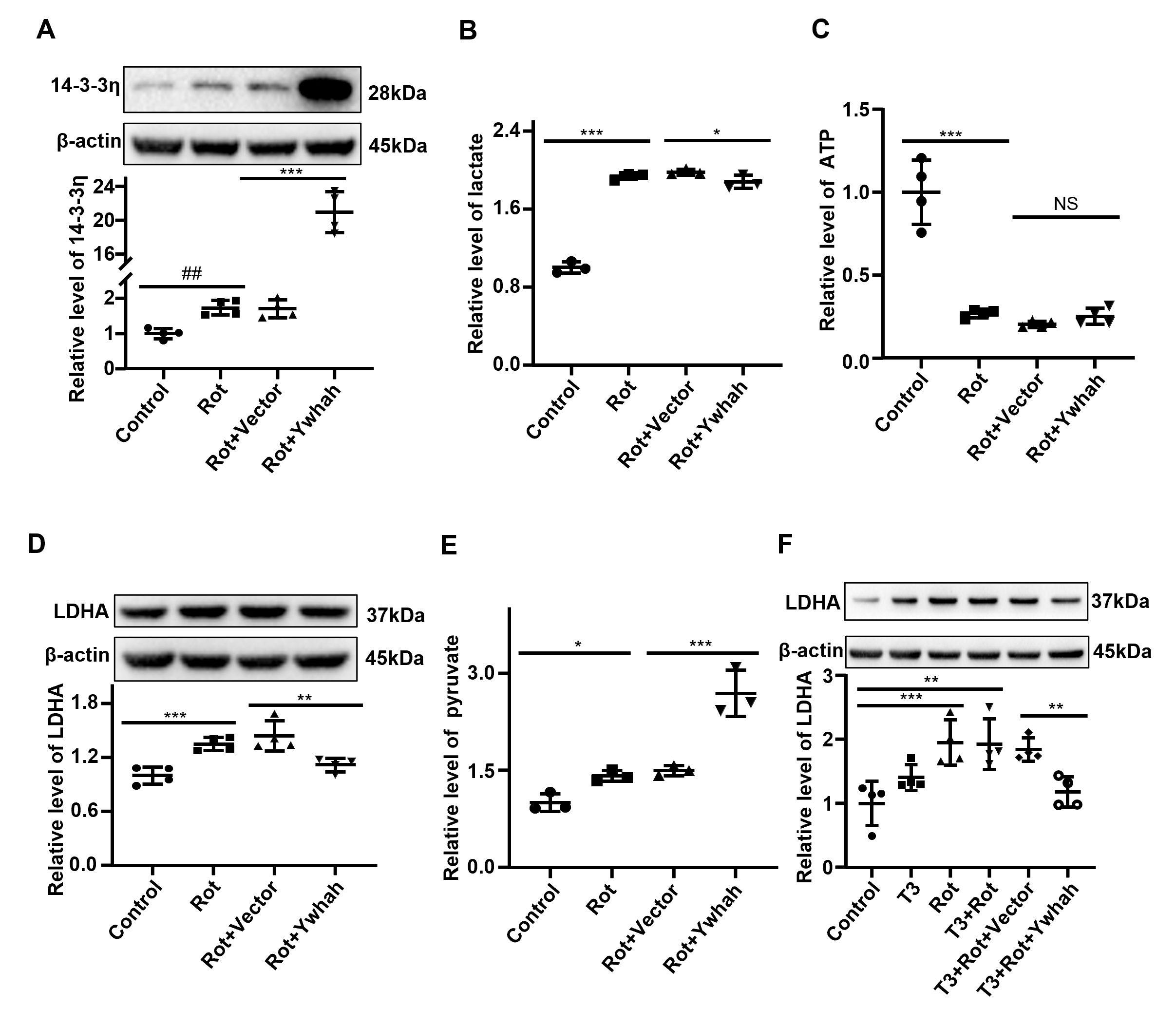

Supplement: S1 Fig — H9C2 cells were transfected with Ywhah or empty Vector plasmid for 8 hours and then stimulated with 100 nM Rotenone (Rot) for 48 hours. A. Representative western blot and semi-quantification statistical data showing the expression of 14-3-3η protein. ## P<0.01 by student t-test. B. Lactate, the product of glycolysis, was detected in the cell culture supernatant. C. ATP level was detected in cell lysate. D. Representative western blot and semi-quantification statistical data showing the expression of LDHA, a glycolysis-related protein. E. Pyruvate, the product of glycolysis, was detected in cell lysate. F. Representative western blot and semi-quantification statistical data showing the level of LDHA in Ywhah or empty Vector plasmid transfected H9C2 cells undergo co-stimulation with T3 and Rotenone (Rot). Data were analyzed by one-way analysis of variance [ANOVA] with LSD posttest (* P<0.05, ** P<0.01, *** P<0.001, NS, no significant), each symbol in graphs A-F representing an independent experiment. (TIF) [file pone.0307696.s001.tif]

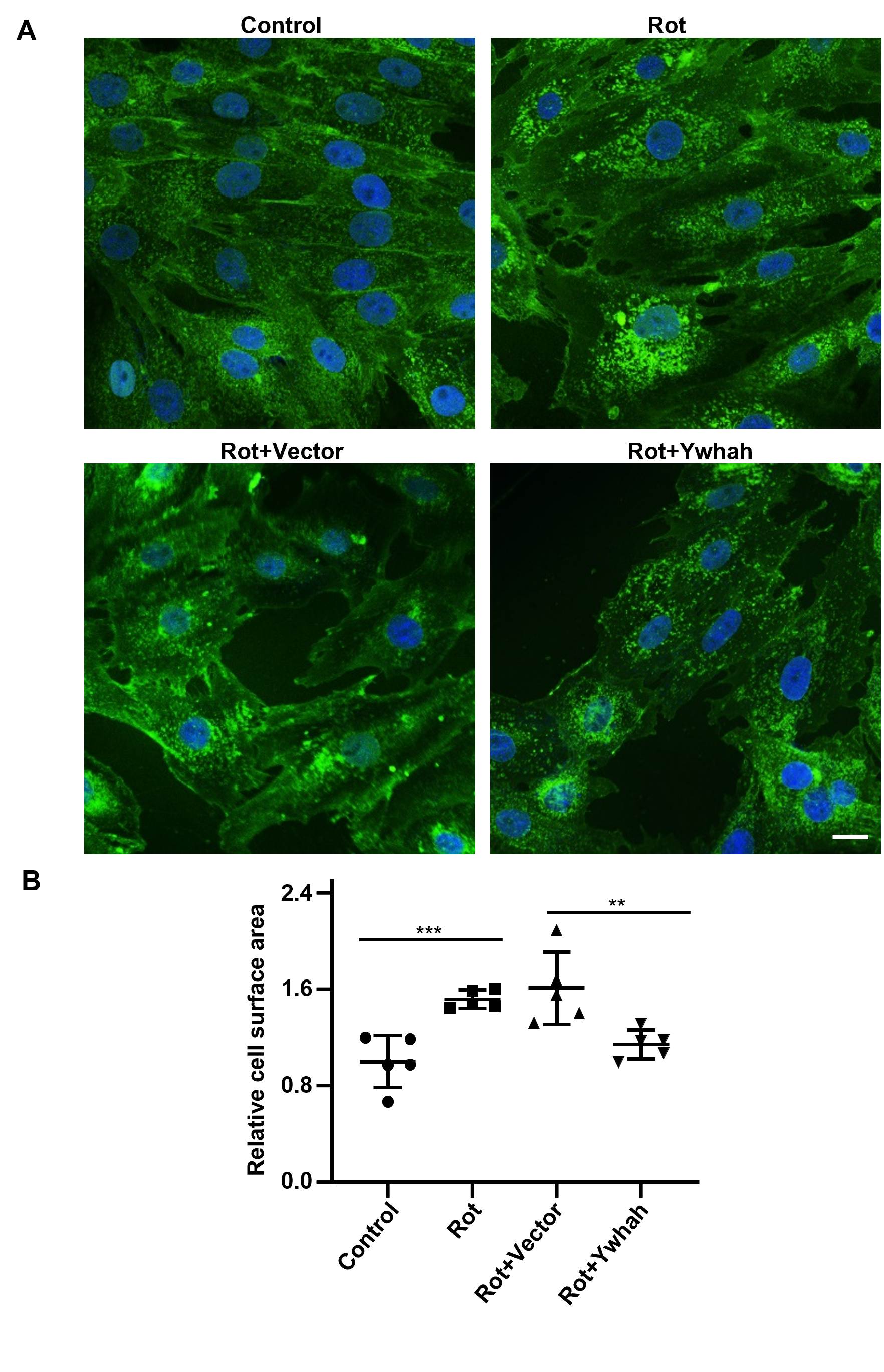

Supplement: S2 Fig — H9C2 cells were transfected with Ywhah or empty Vector plasmid for 8 hours and then stimulated with 100 nM Rotenone (Rot) for 48 hours. A. and B. Representative WGA staining images (A) show the cell surface area and their statistical analysis (B). Bar = 10 μm. Data were analyzed by one-way analysis of variance [ANOVA] with LSD posttest (** P<0.01, *** P<0.001), each symbol representing a random microscopic field. (TIF) [file pone.0307696.s002.tif]

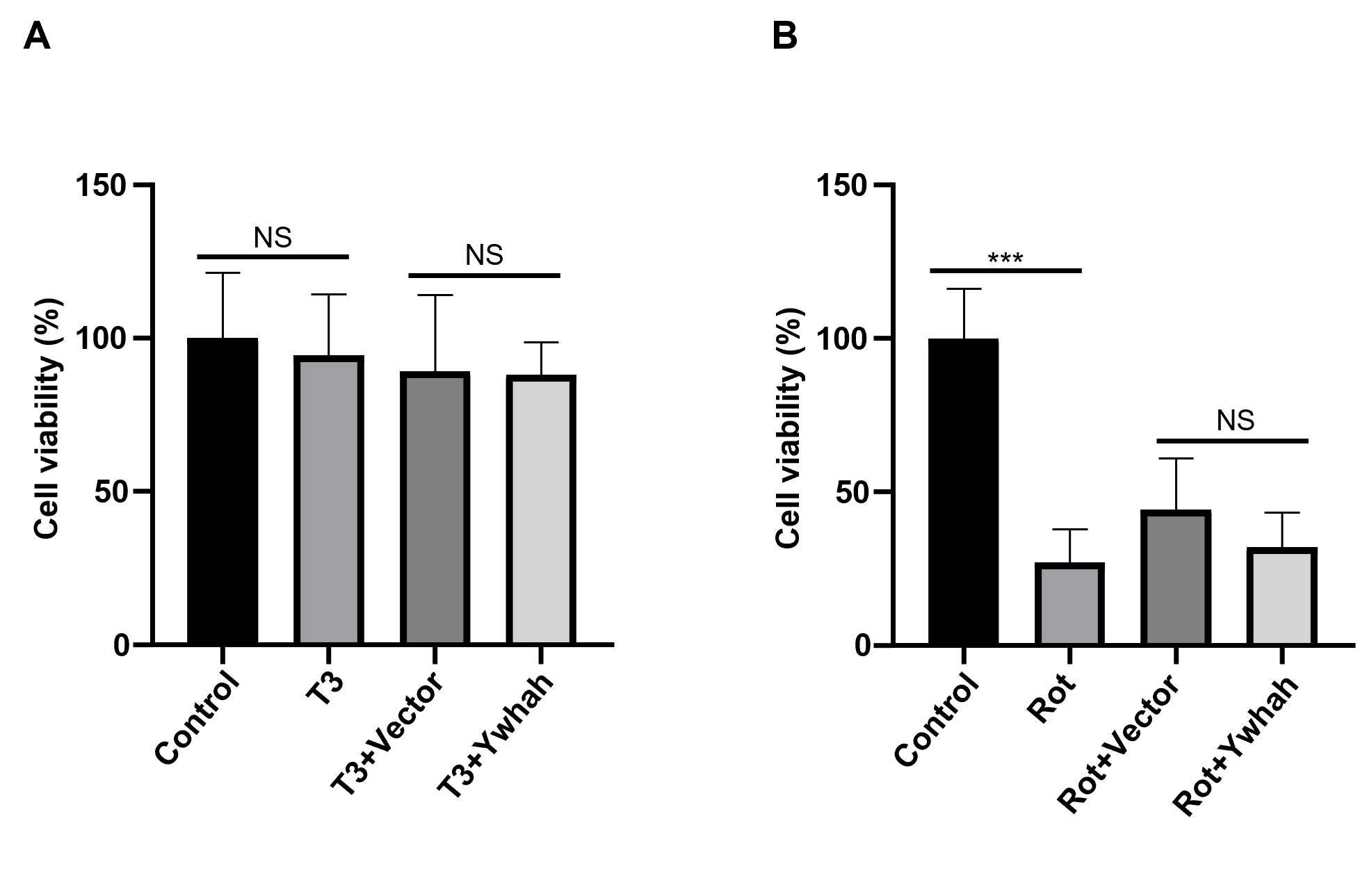

Supplement: S3 Fig — (A-B) H9C2 cells were transfected with Ywhah or empty Vector plasmid for 8 hours, the cell viability was detected by CCK-8 assay after further 48 hours stimulation with T3 (A) or rotenone (Rot) (B). Data were analyzed by one-way analysis of variance [ANOVA] with LSD posttest (*** P<0.001, NS, no significant). (TIF) [file pone.0307696.s003.tif]

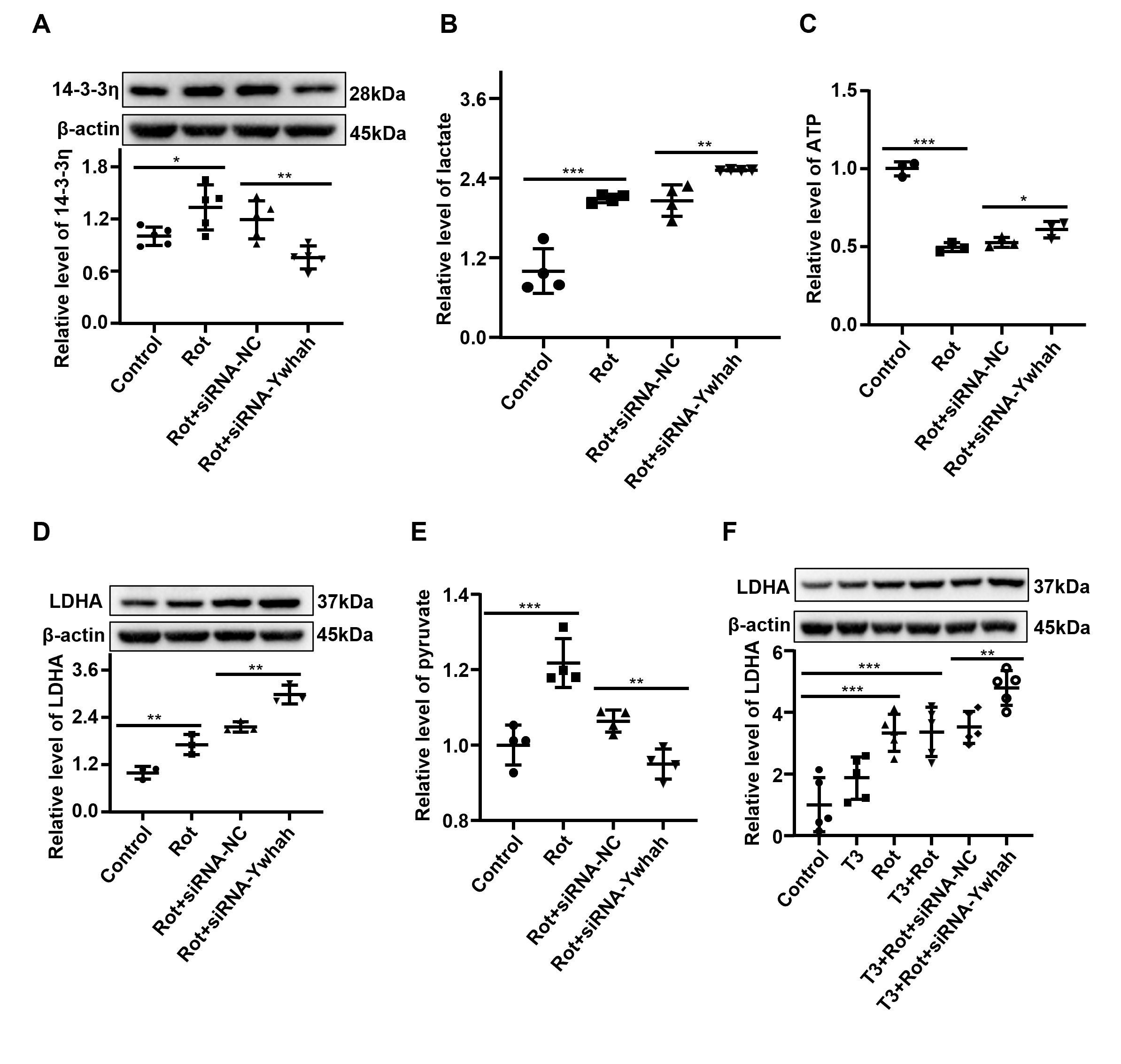

Supplement: S4 Fig — H9C2 cells were transfected with siRNA-Ywhah or NC for 8 hours and then stimulated with 100 nM Rotenone (Rot) for 48 hours. A. Representative western blot and semi-quantification statistical data showing the expression of 14-3-3η protein. B. Lactate, the product of glycolysis, was detected in the cell culture supernatant. C. ATP level was detected in cell lysate. D. Representative western blot and semi-quantification statistical data showing the expression of LDHA, a glycolysis-related protein. E. Pyruvate, the product of glycolysis, was detected in cell lysate. F. Representative western blot and semi-quantification statistical data showing the level of LDHA in siRNA-Ywhah or NC transfected H9C2 cells undergo co-stimulation with T3 and Rotenone (Rot). Data were analyzed by one-way analysis of variance [ANOVA] with LSD posttest (* P<0.05, ** P<0.01, *** P<0.001), each symbol in graphs A-F representing an independent experiment. (TIF) [file pone.0307696.s004.tif]

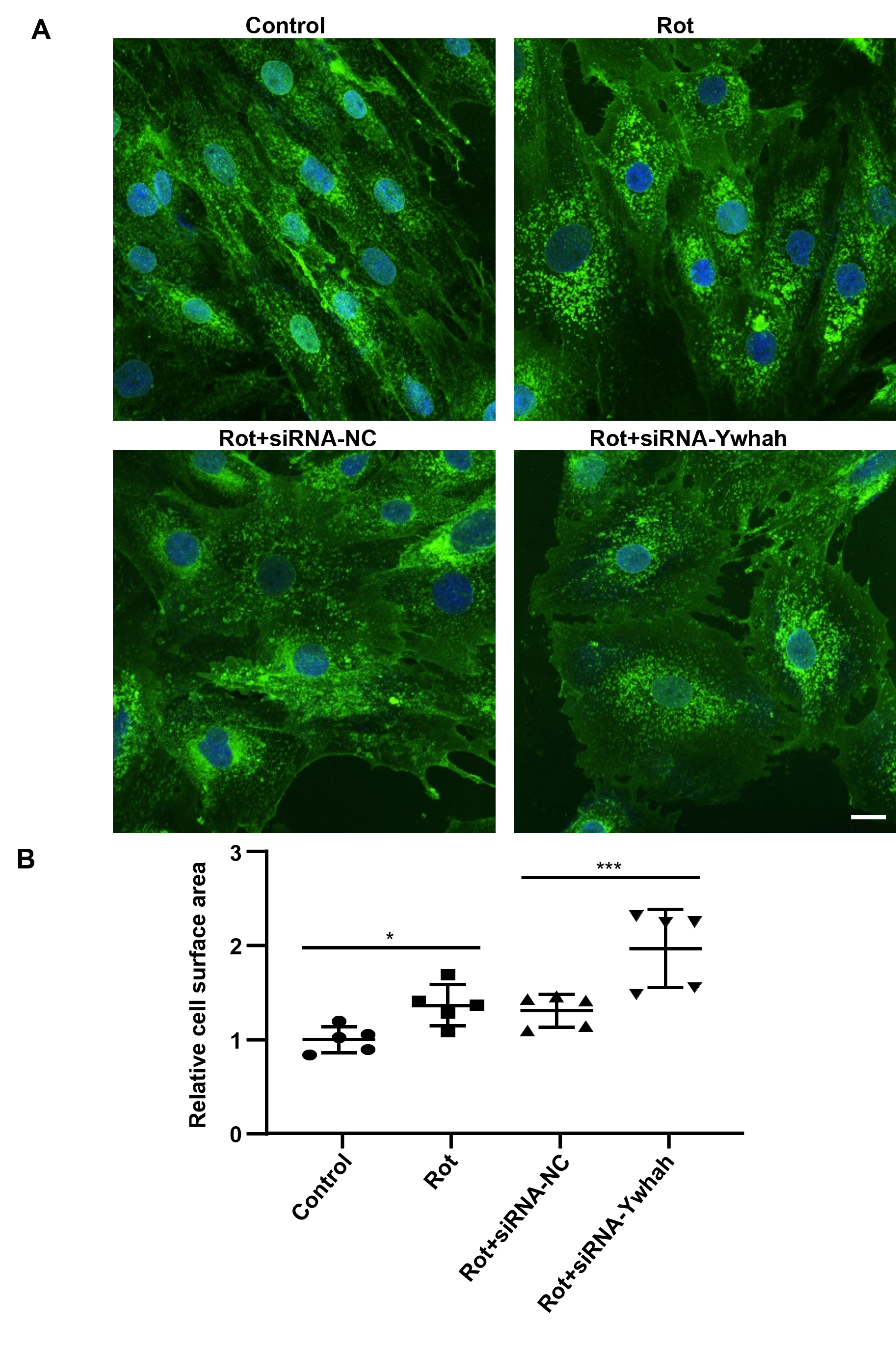

Supplement: S5 Fig — H9C2 cells were transfected with siRNA-Ywhah or NC for 8 hours and then stimulated with 100 nM Rotenone (Rot) for 48 hours. A. and B. Representative WGA staining images (A) show the cell surface area and their statistical analysis (B). Bar = 10 μm. Data were analyzed by one-way analysis of variance [ANOVA] with LSD posttest (* P<0.05, *** P<0.001), each symbol representing a random microscopic field. (TIF) [file pone.0307696.s005.tif]

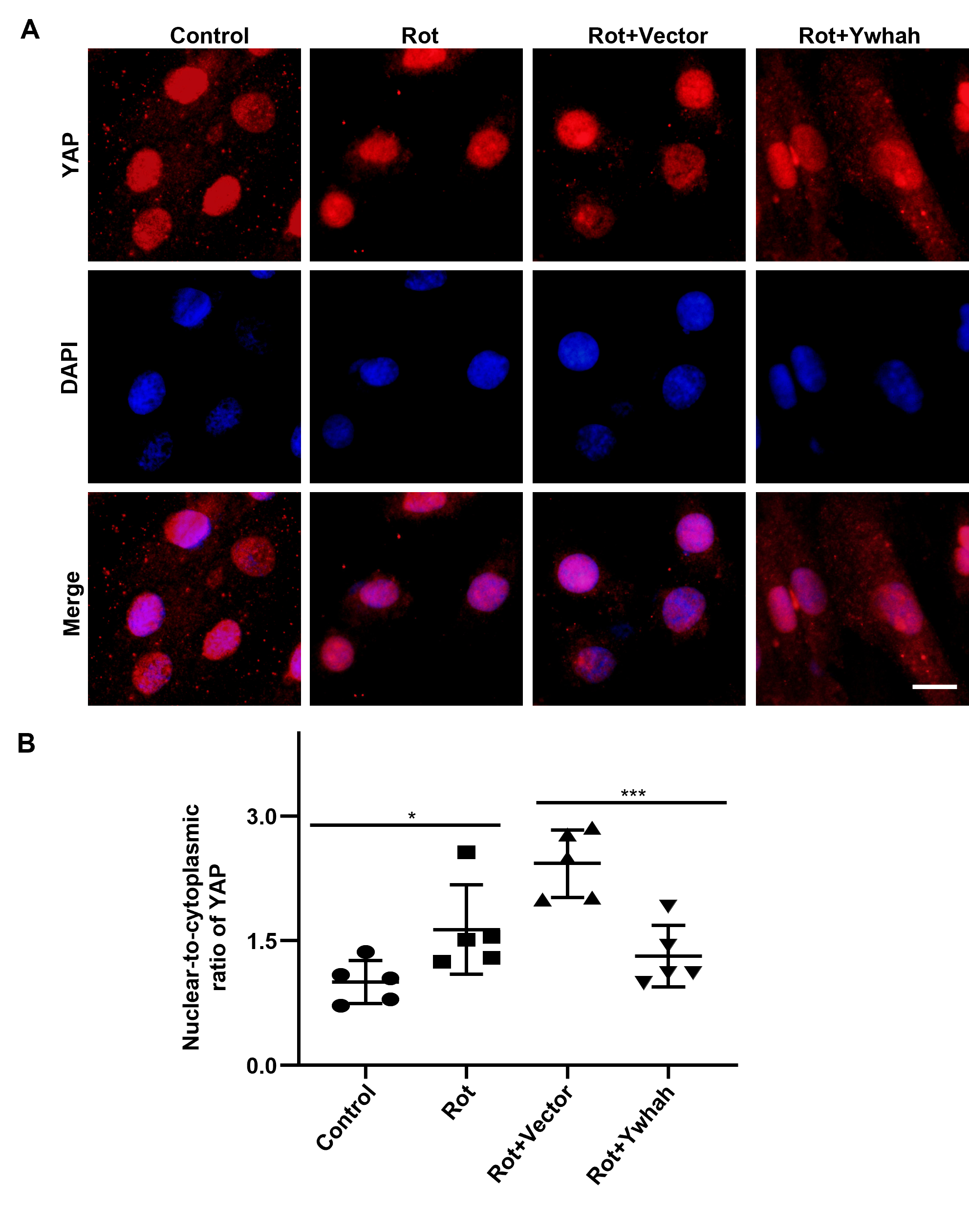

Supplement: S6 Fig — H9C2 cells were transfected with Ywhah or empty Vector plasmid for 8 hours and then stimulated with 100 nM Rotenone (Rot) for 48 hours. A. and B. Detection the nuclear translocation of YAP by immunofluorescence staining (A) and their statistical analysis (B). Bar = 20 μm. Data were analyzed by one-way analysis of variance [ANOVA] with LSD posttest (* P<0.05, *** P<0.001), each symbol representing a random microscopic field. (TIF) [file pone.0307696.s006.tif]

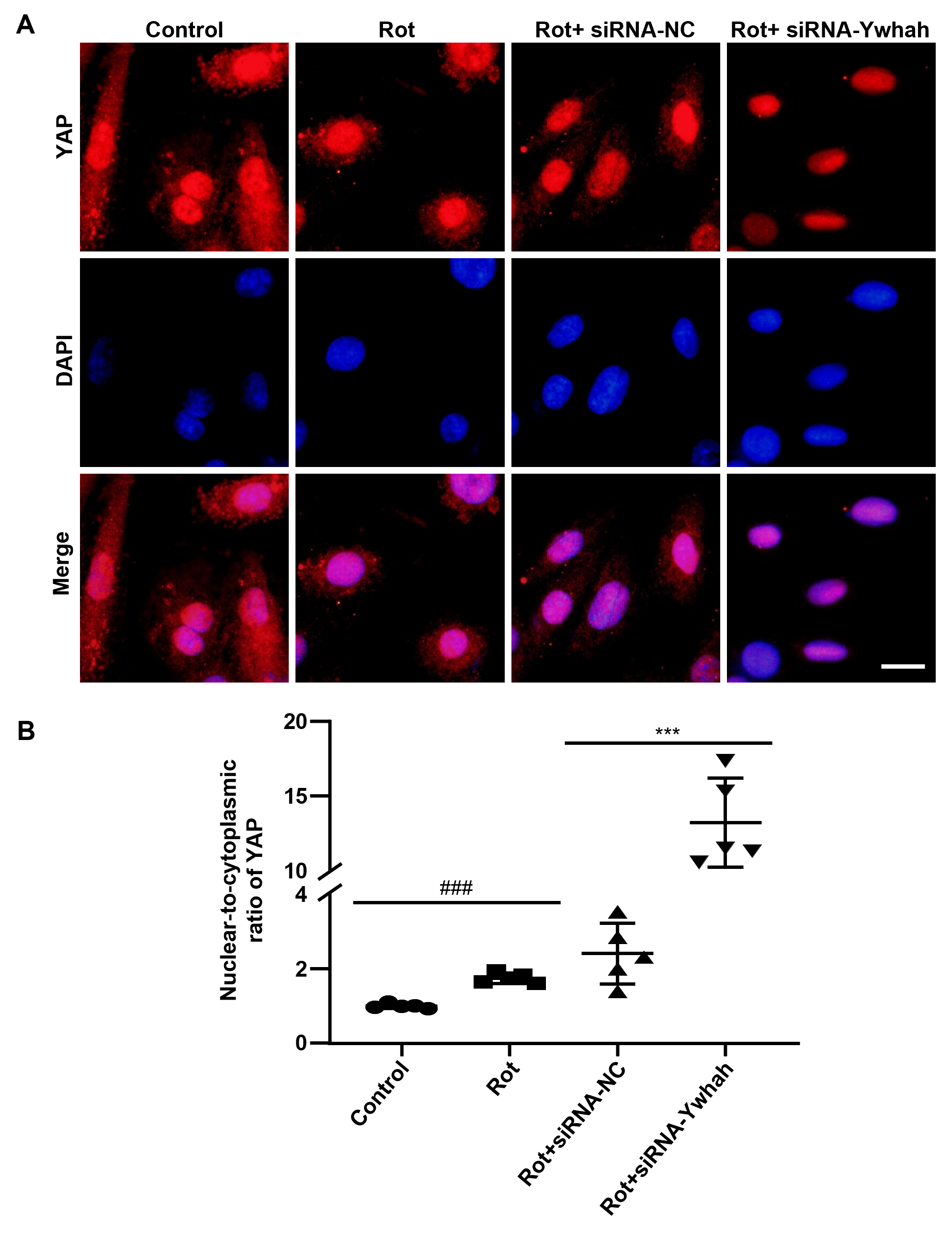

Supplement: S7 Fig — H9C2 cells were transfected with siRNA-Ywhah or NC for 8 hours and then stimulated with 100 nM Rotenone (Rot) for 48 hours. A. and B. Detection the nuclear translocation of YAP by immunofluorescence staining (A) and their statistical analysis (B). Bar = 20 μm. Data were analyzed by one-way analysis of variance [ANOVA] with LSD posttest (*** P<0.001), each symbol representing a random microscopic field. ### P<0.001 by student t-test. (TIF) [file pone.0307696.s007.tif]
