# Supplementary material for: Regulation of H9C2 cell hypertrophy by 14-3-3η via inhibiting glycolysis
Source: PLoS One. 2024 Jul 22;19(7):e0307696. doi: 10.1371/journal.pone.0307696 (PMC11262655; doi:10.1371/journal.pone.0307696)

Fig. 1 A-B

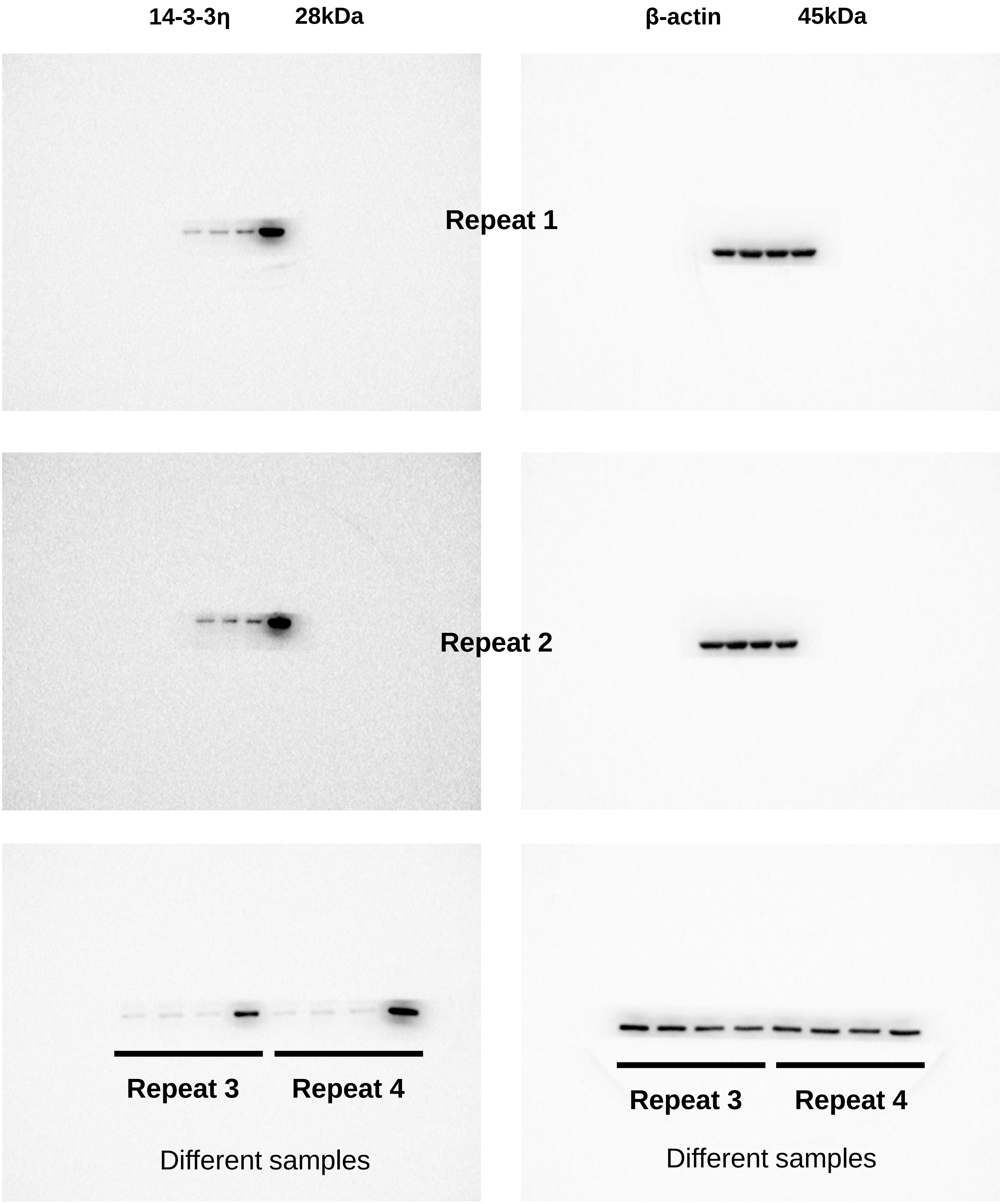

14-3-3η
28kDa
β-actin
45kDa
Repeat 1
Repeat 2
Repeat 3
Repeat 4
Different samples
Repeat 3
Repeat 4
Different samples

Fig. 1 A-C

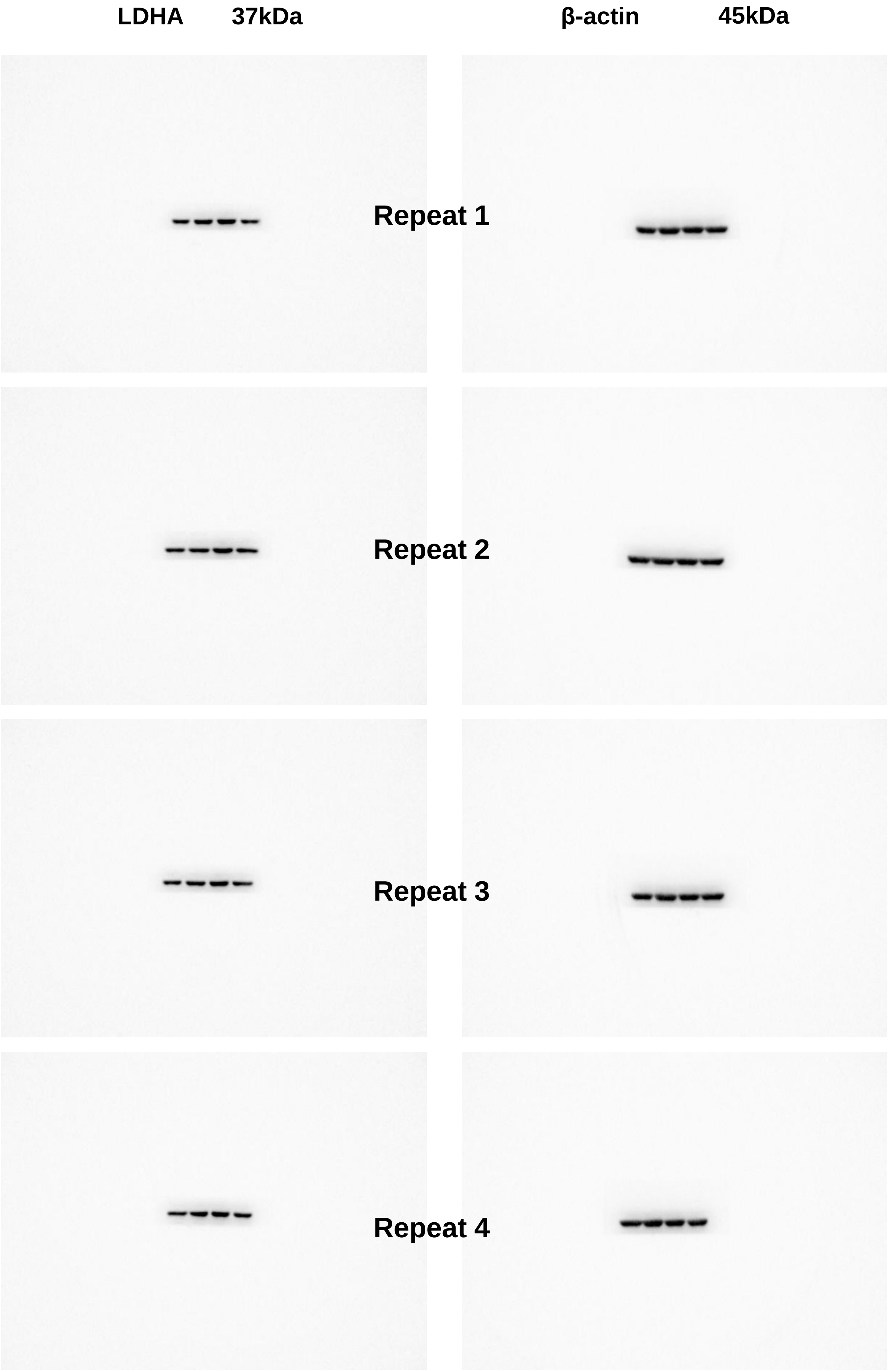

Fig. 2 A

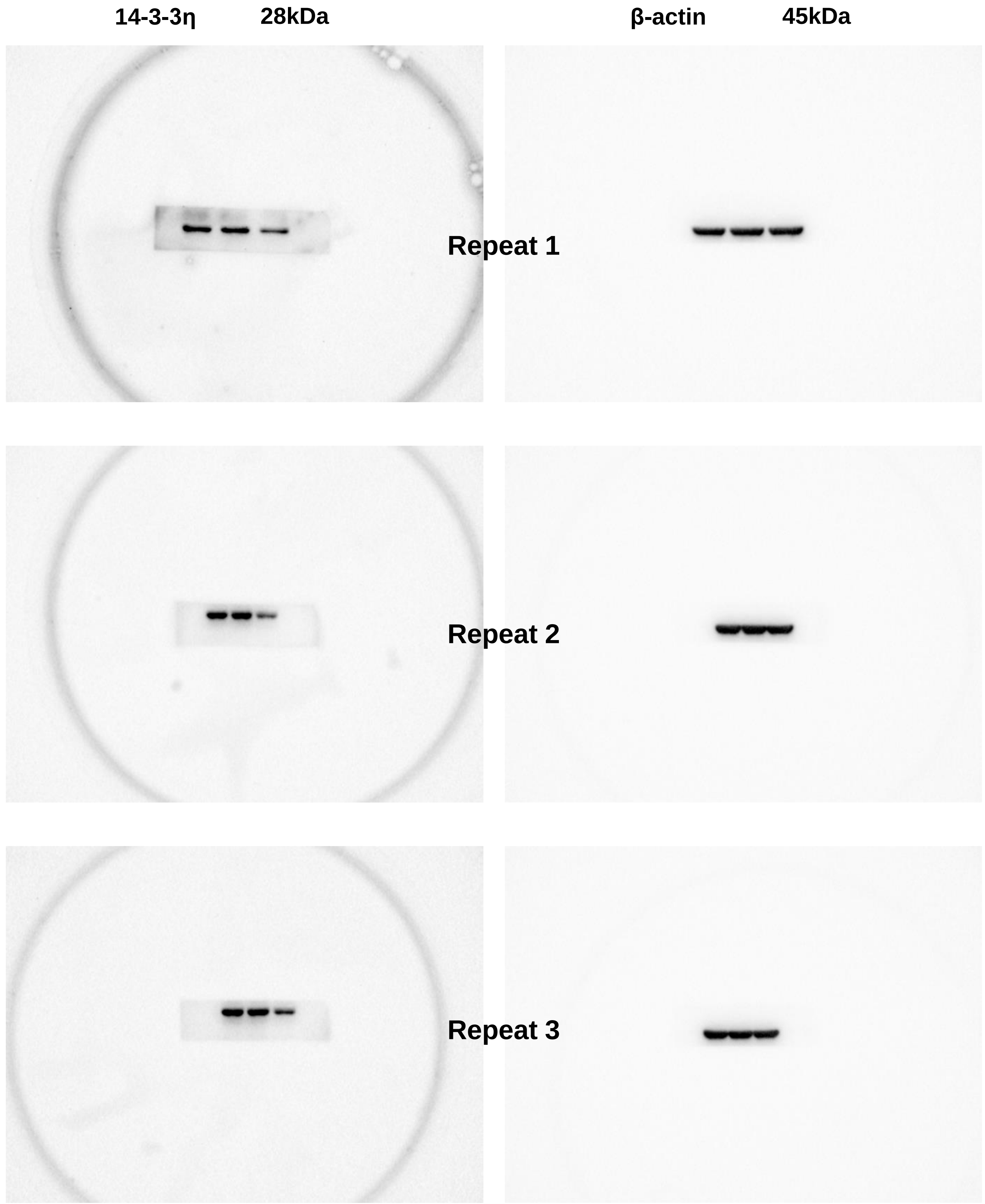

Fig. 2 B

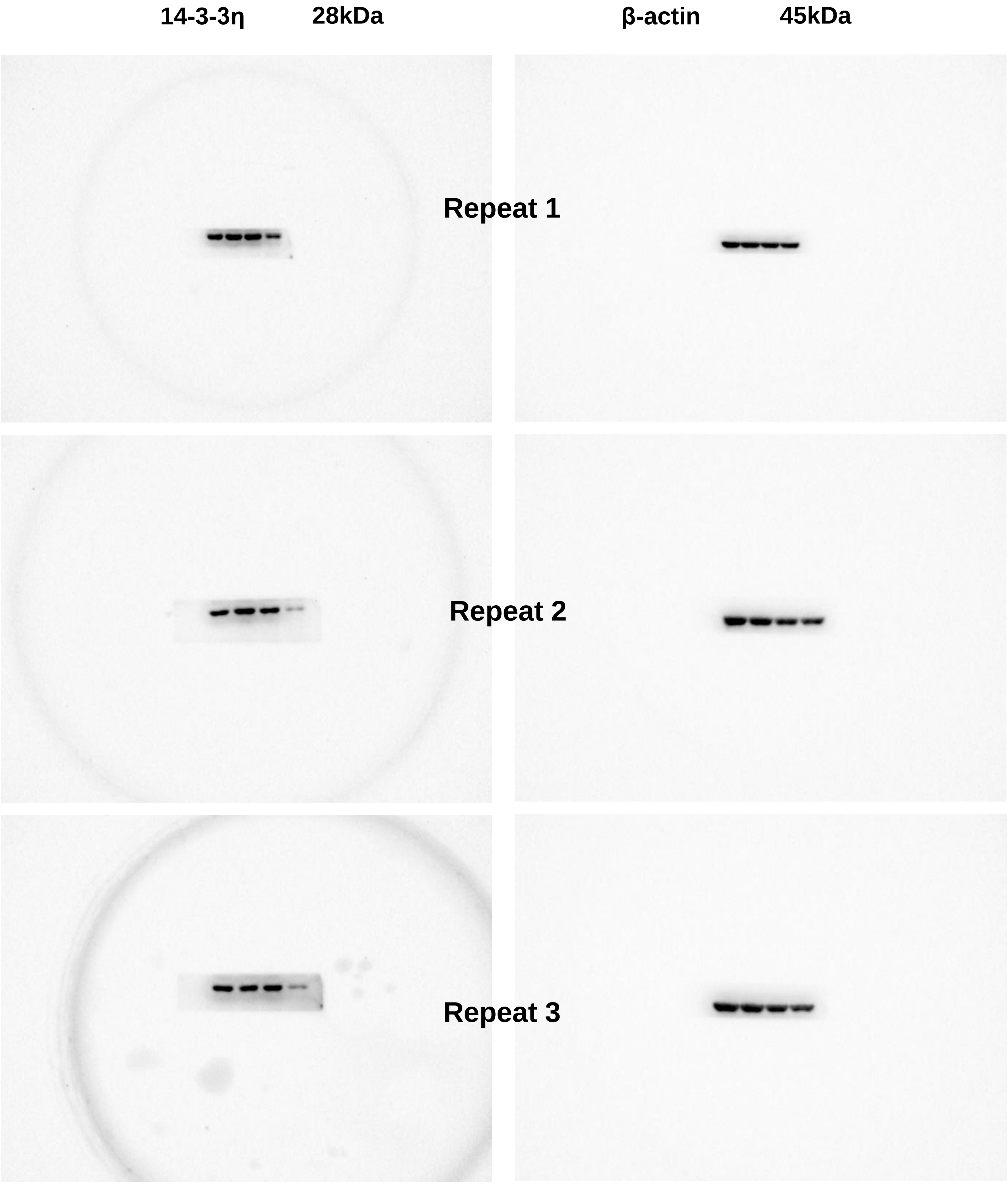

Fig. 2 B

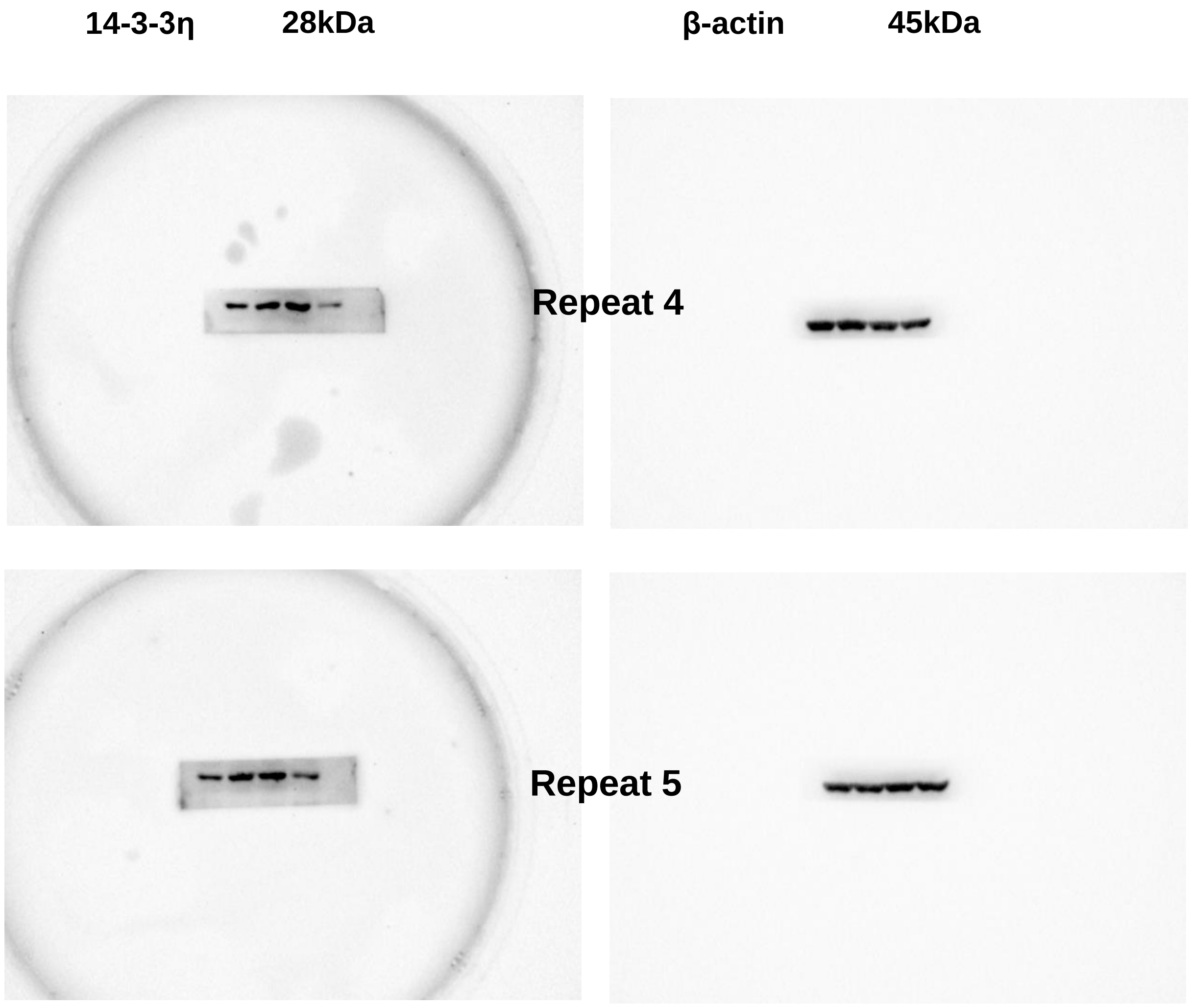

Fig. 2 D

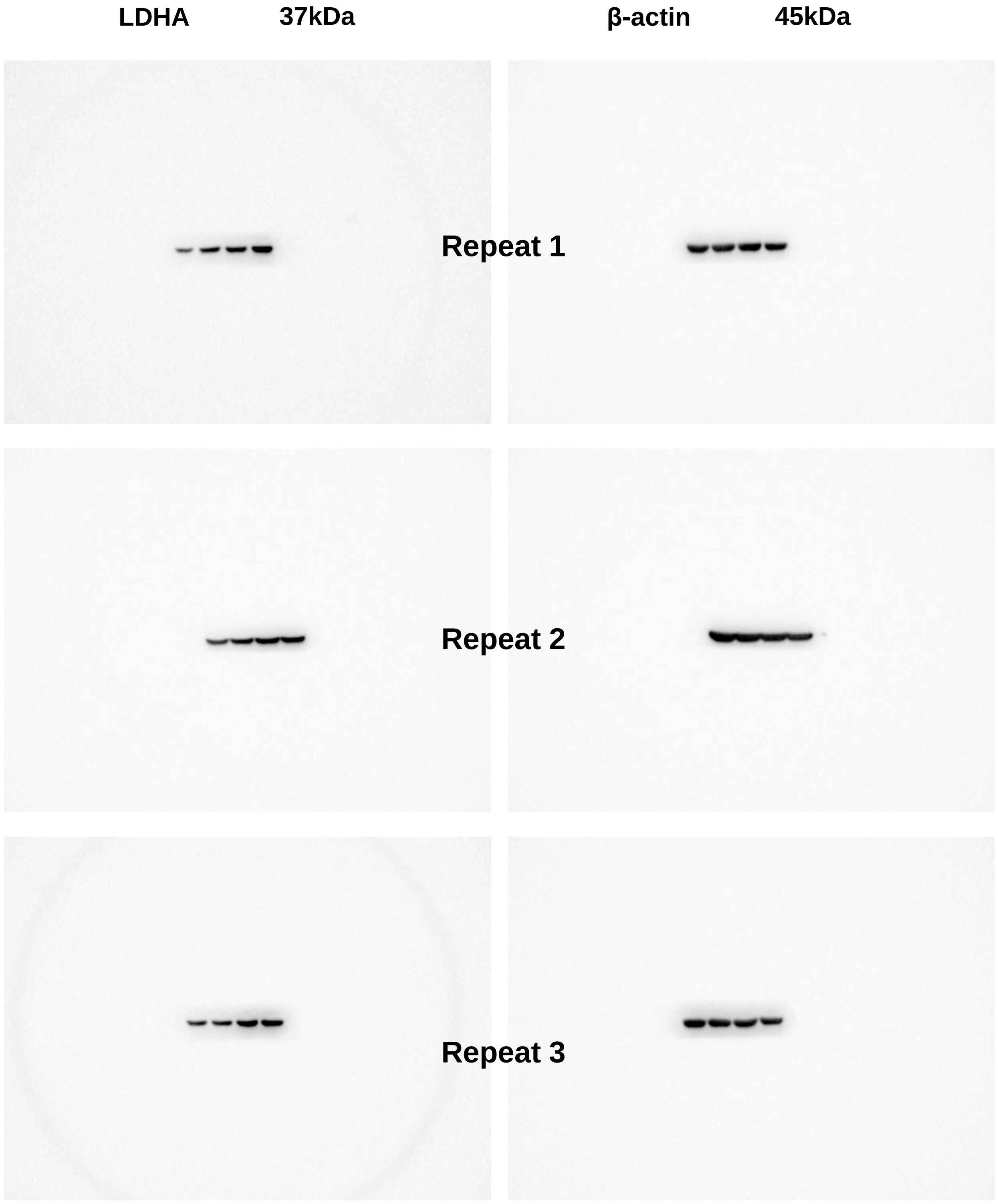

Fig. 2 D

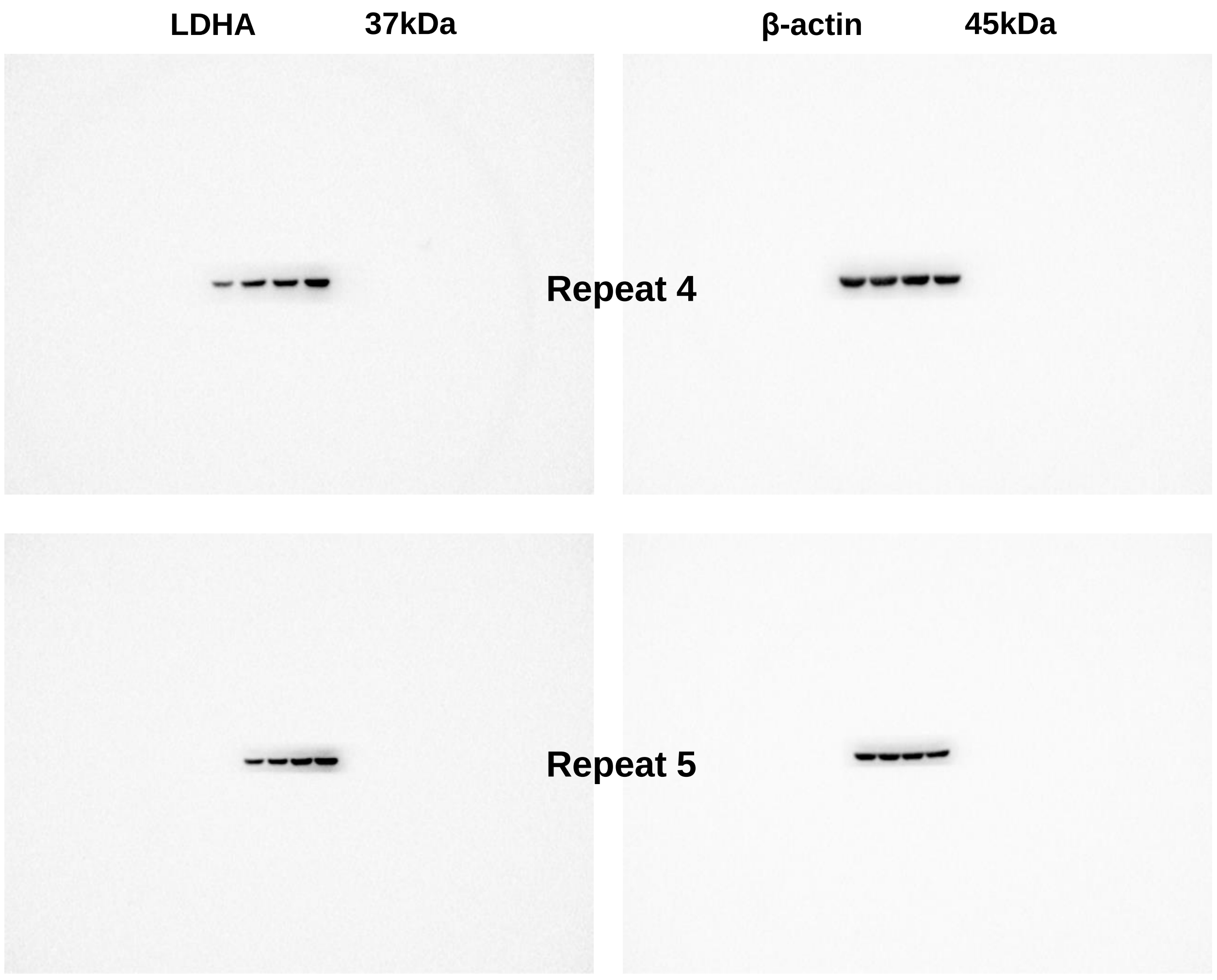

Fig. 3 A

YAP 65kDa

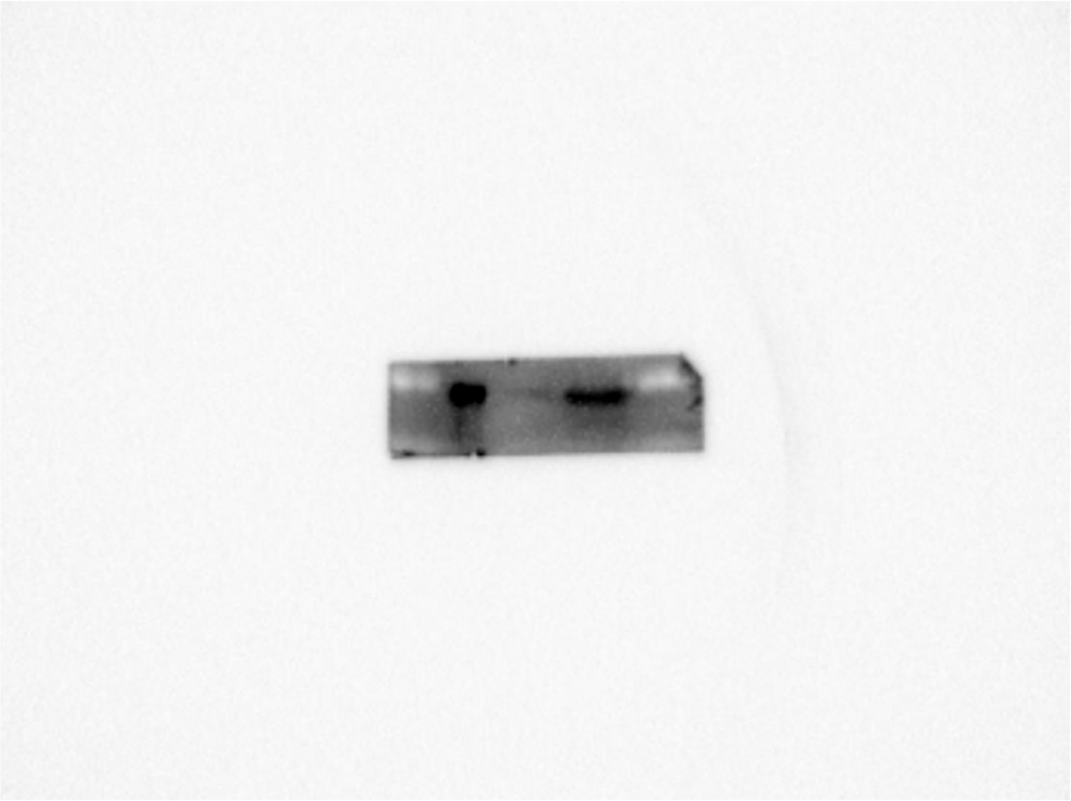

14-3-3η 28kDa

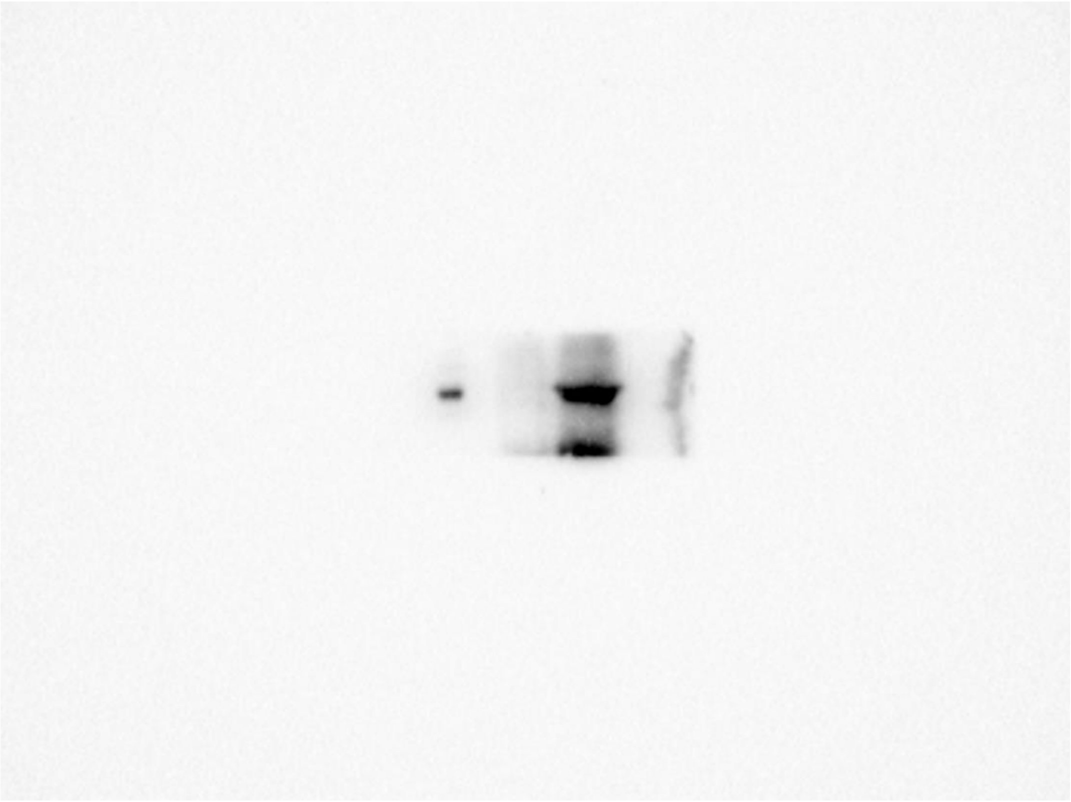

Fig. 3 B

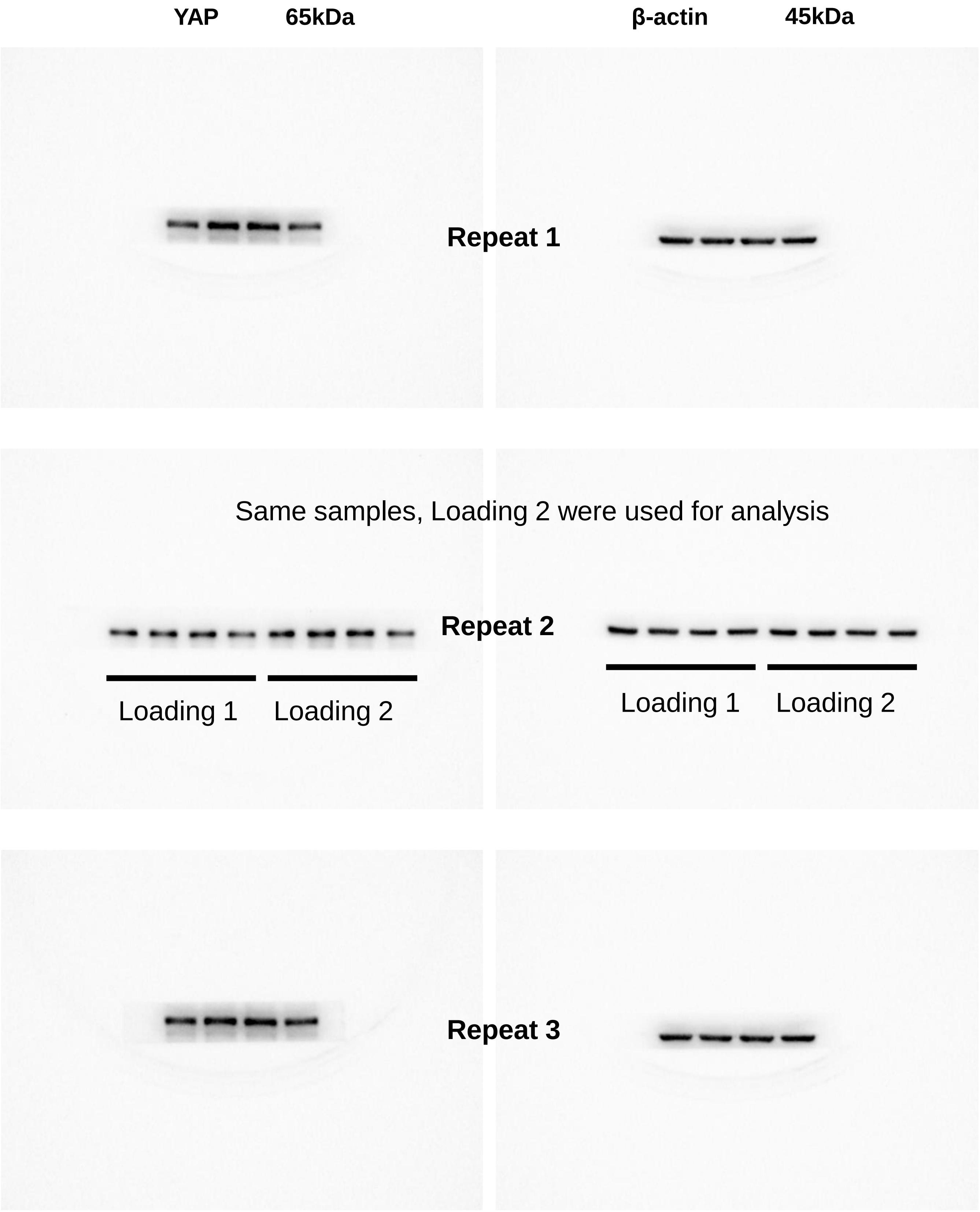
YAP
65kDa
β-actin
45kDa
Repeat 1
Same samples, Loading 2 were used for analysis
Repeat 2
Loading 1
Loading 2
Loading 1
Loading 2
Repeat 3

Fig. 3 C

YAP 65kDa

β-actin 45kDa

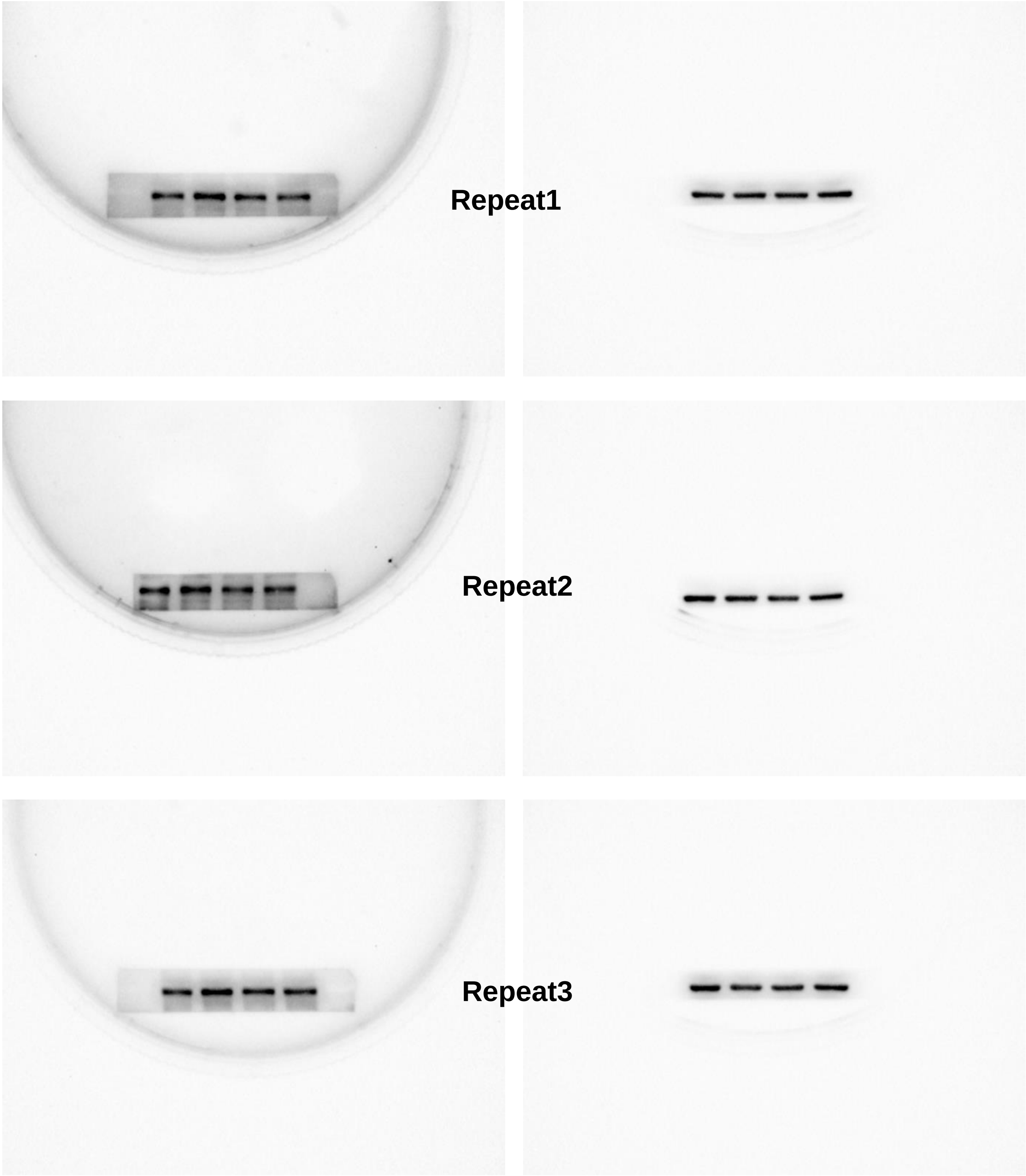

Fig. 3 D

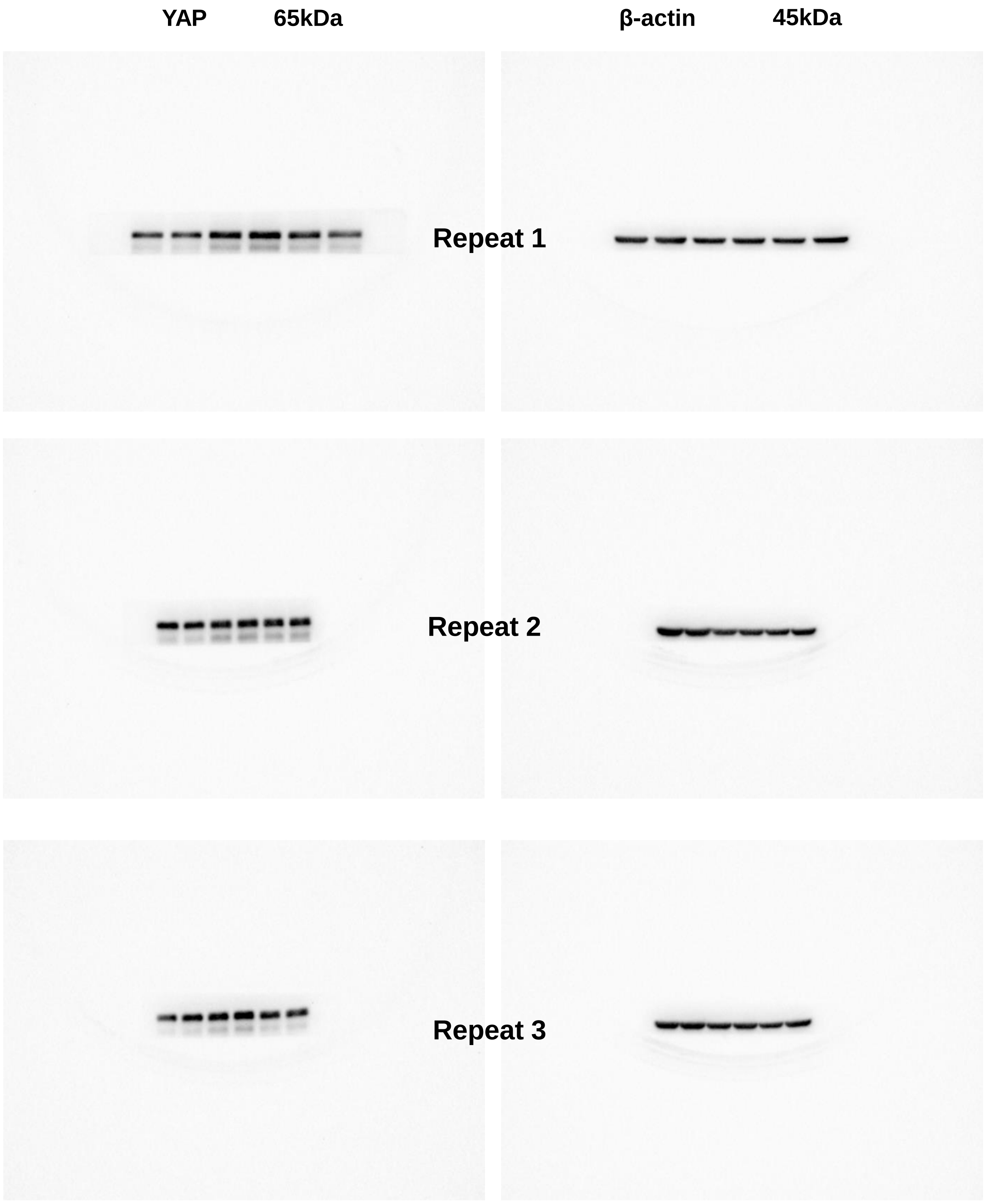

Fig. 3 D

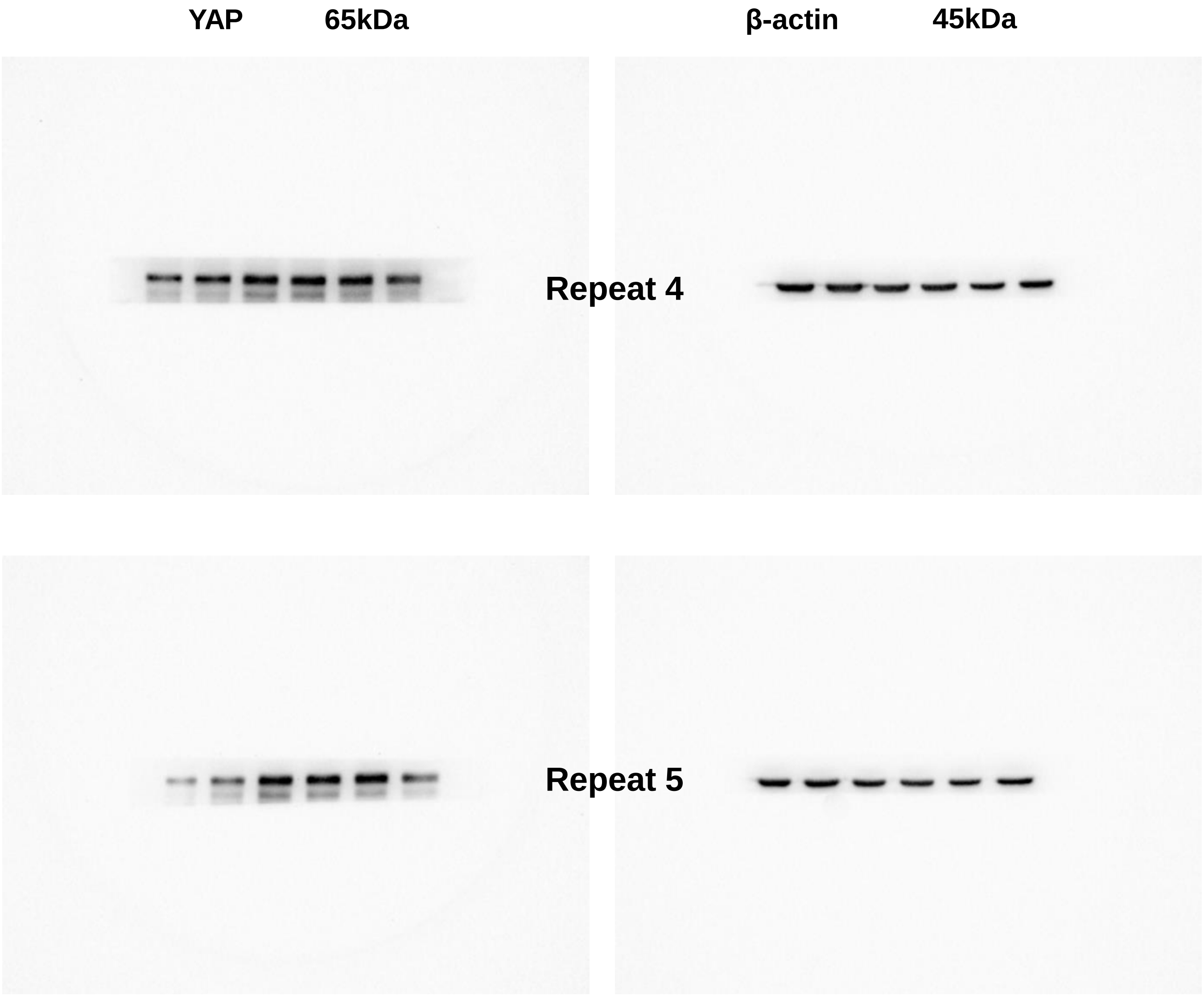

Fig. 3 E

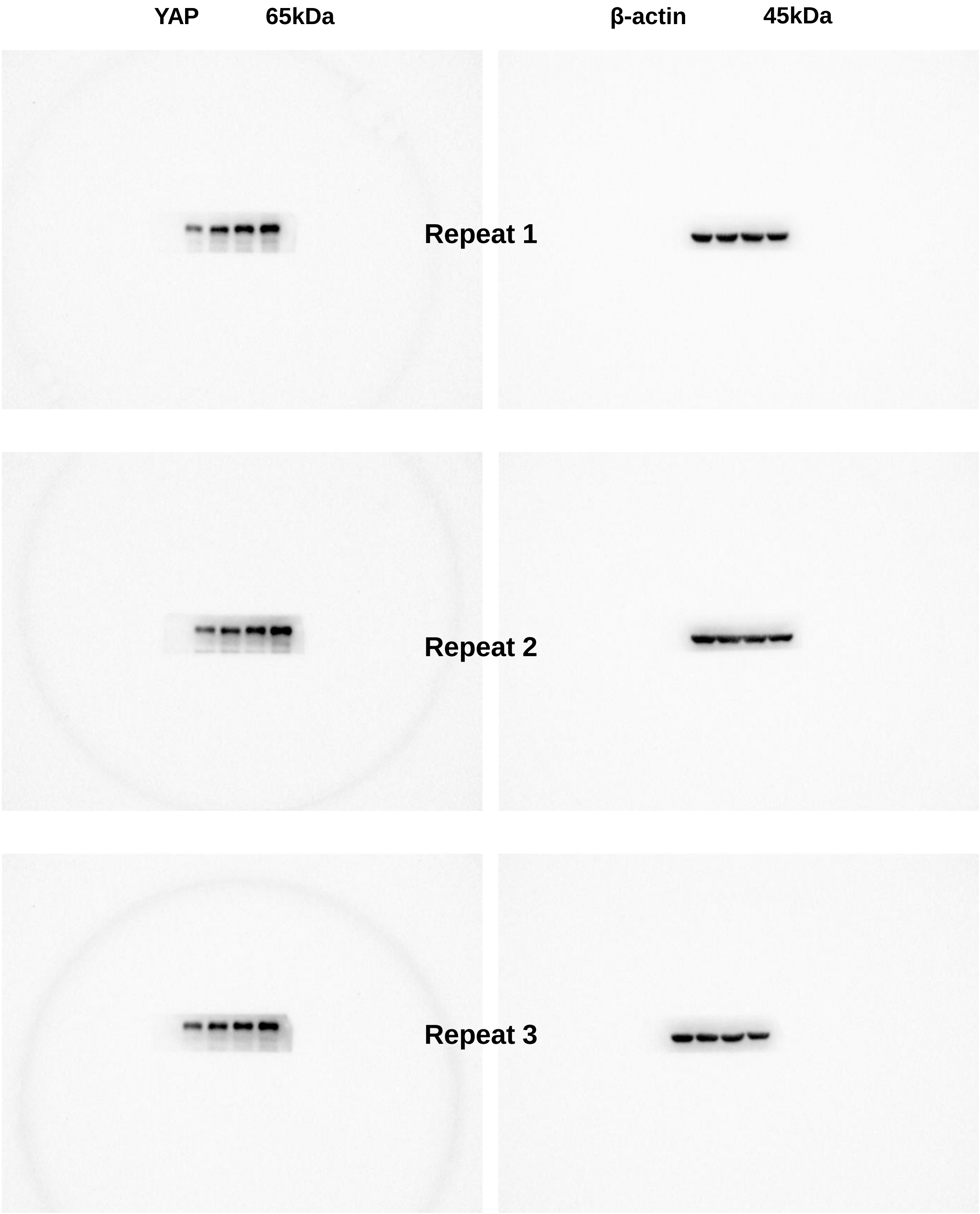

Fig. 3 F

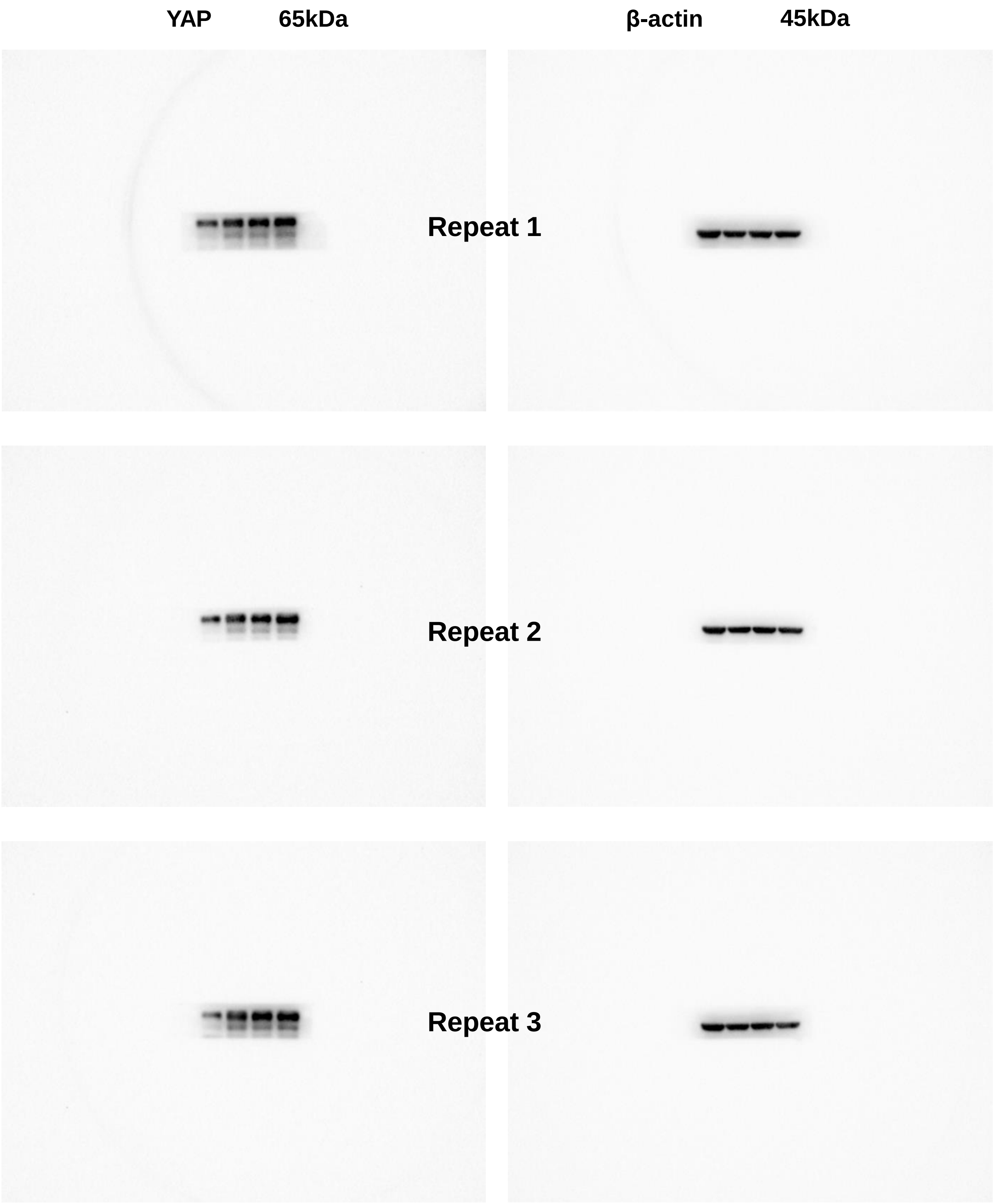

Fig. 3 F

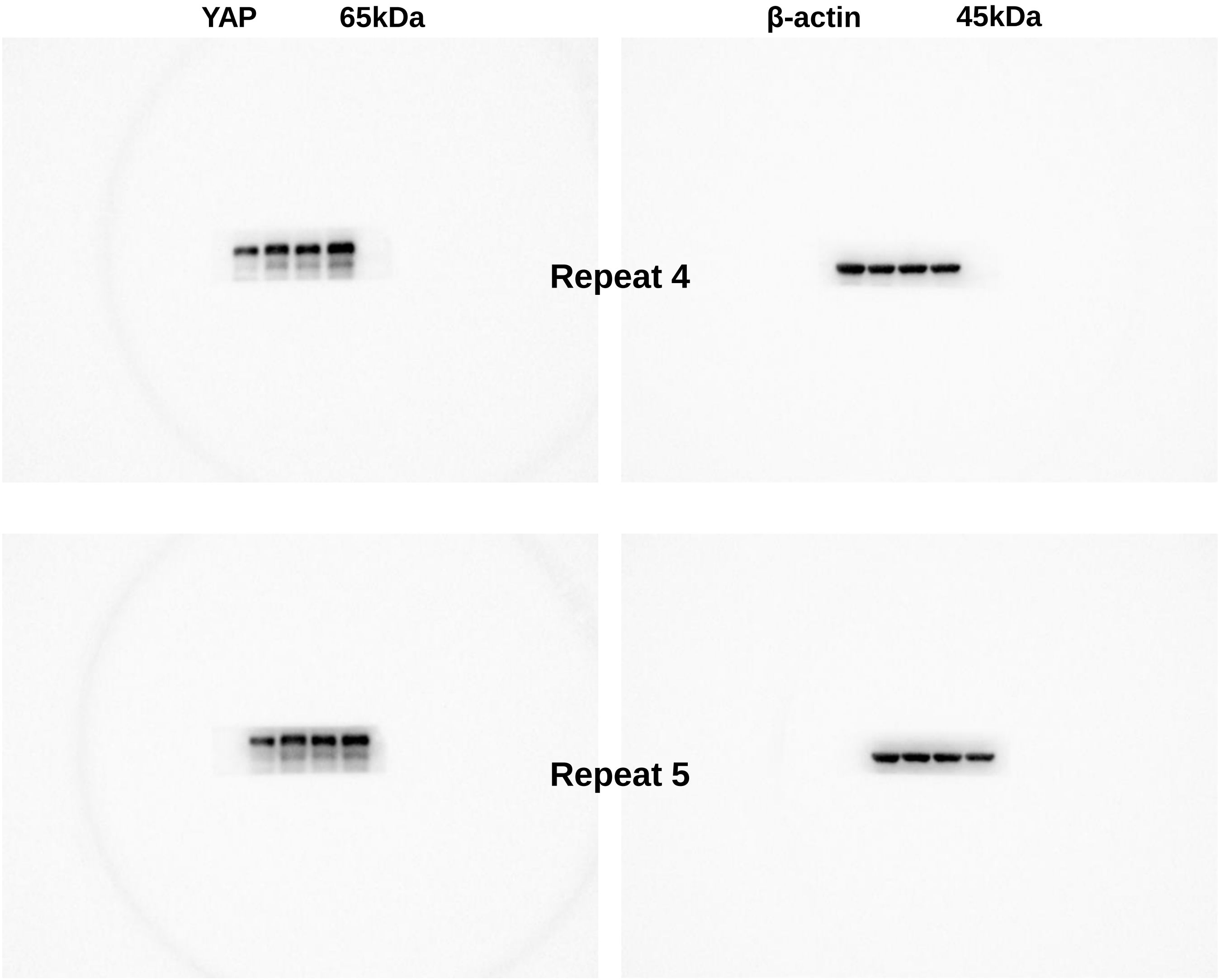

Fig. 3 G

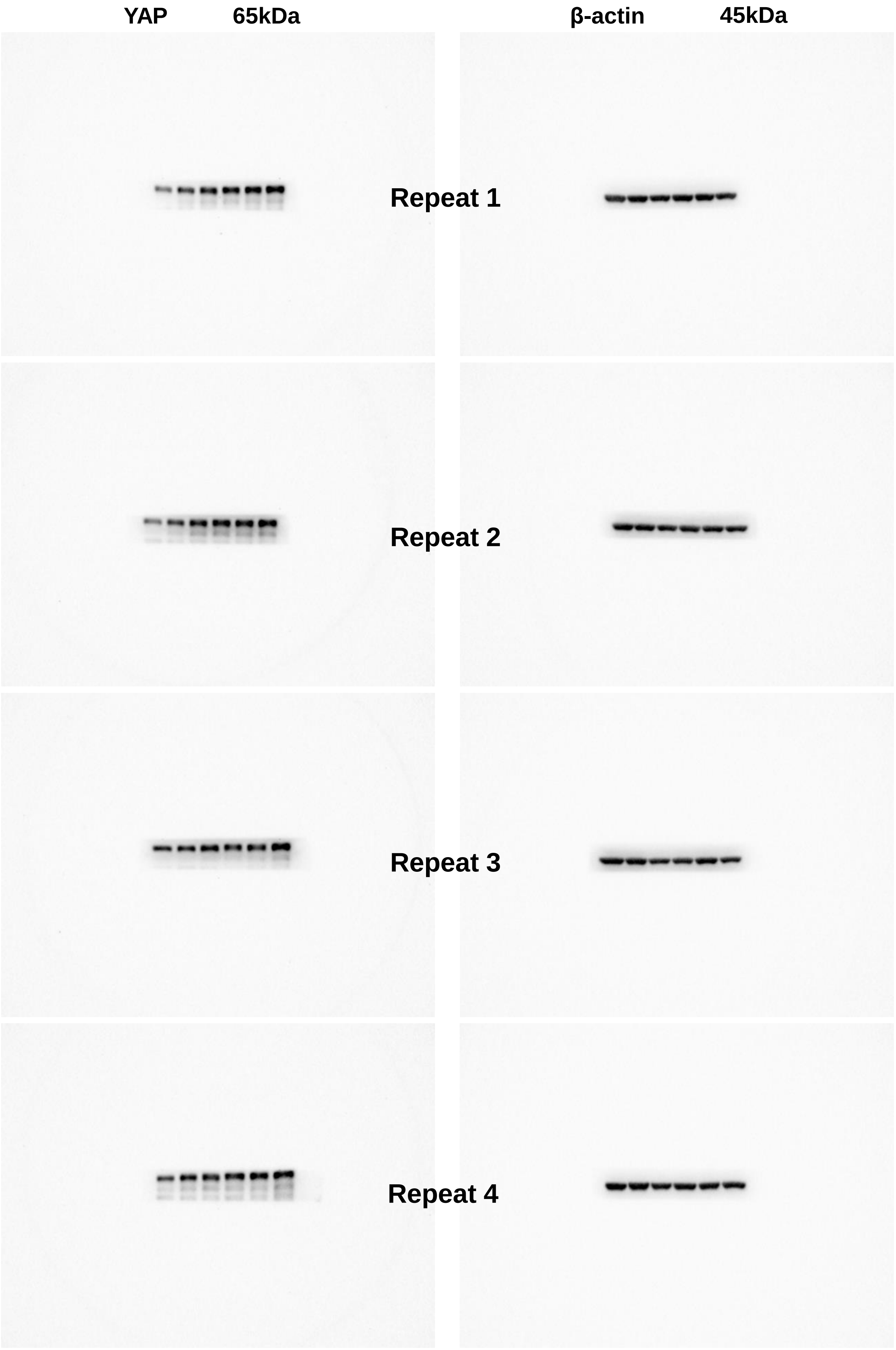

Fig. 4 B **Cytoplasm**

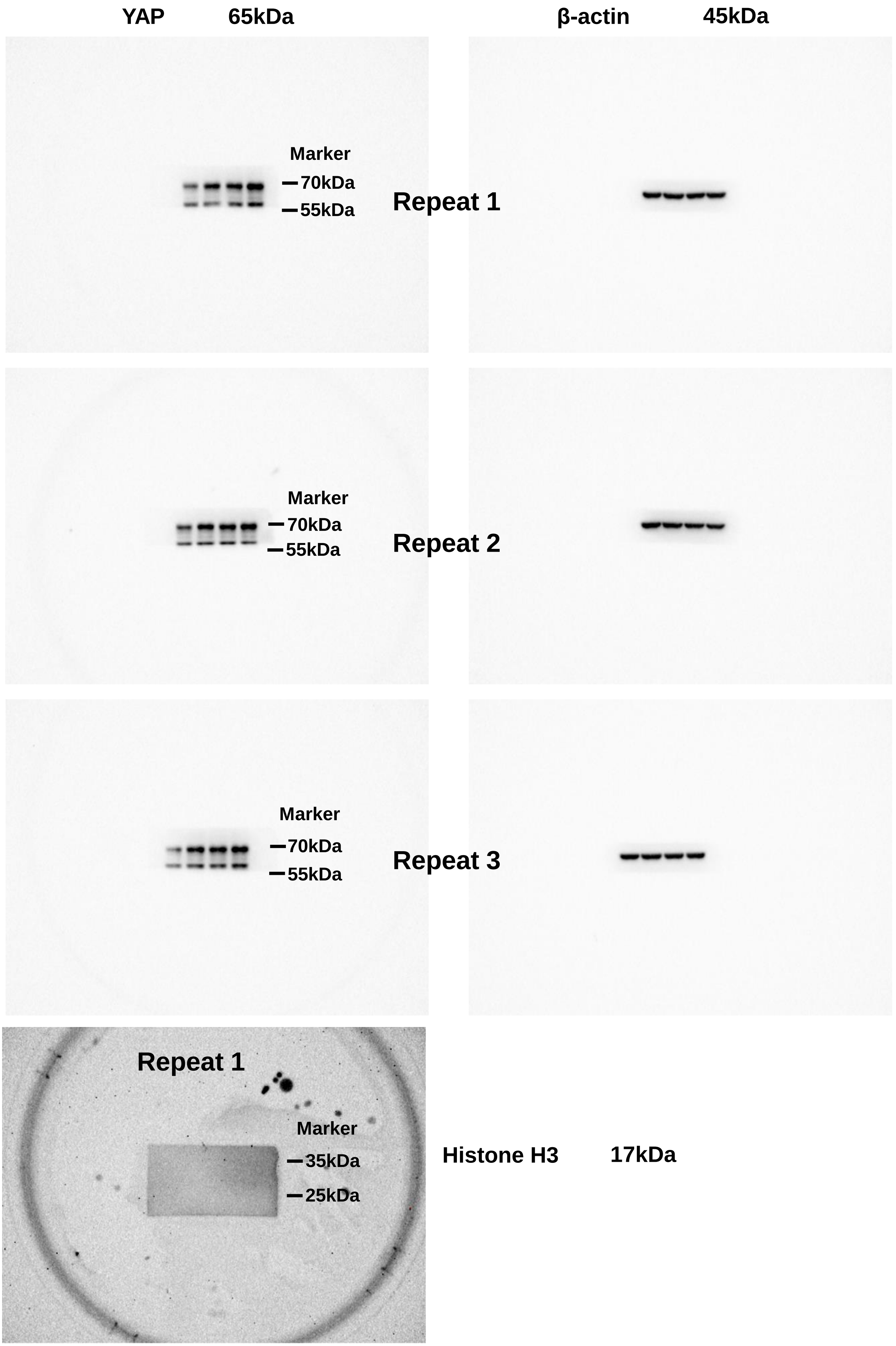

Fig. 4 B **Nucleus**

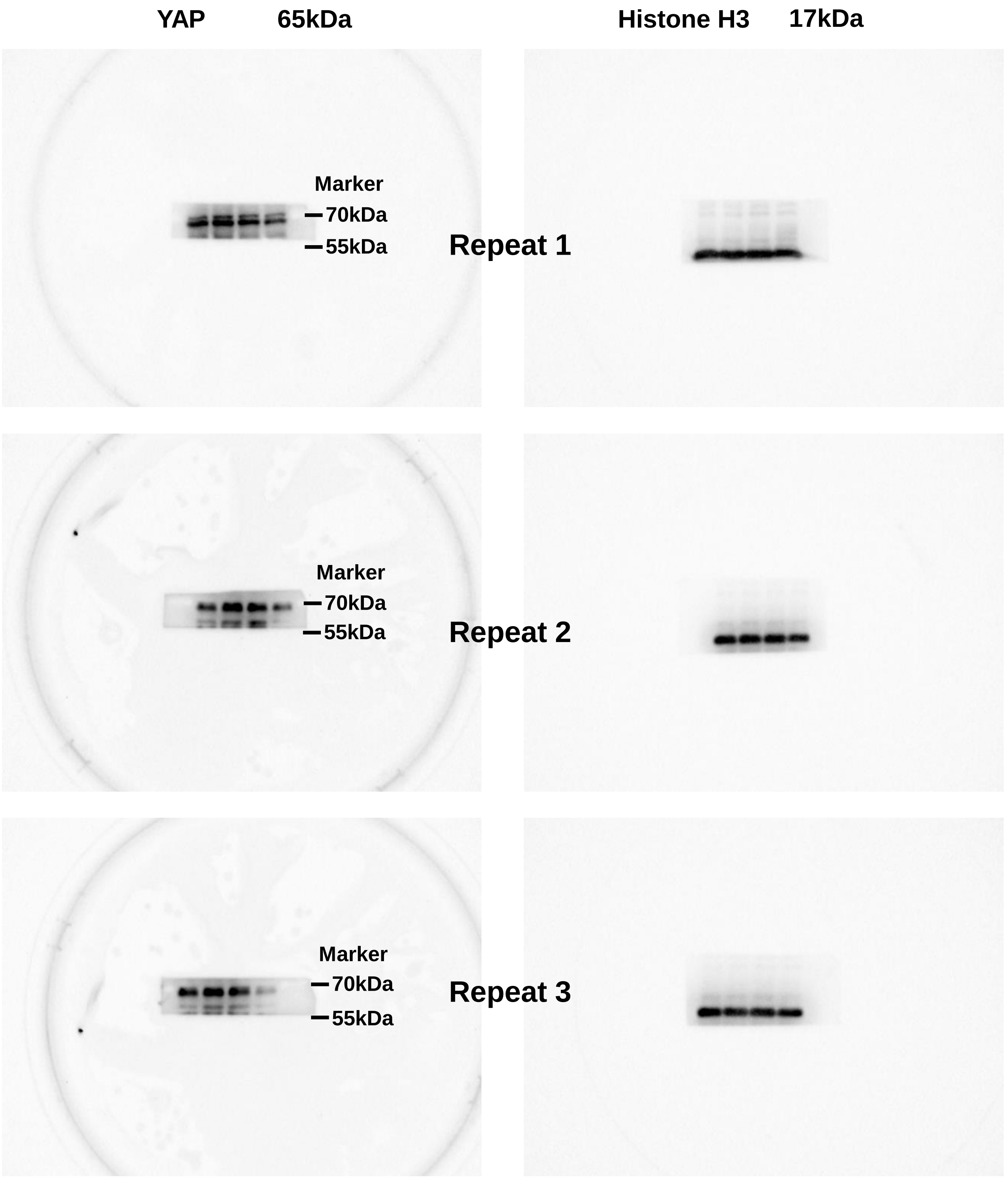

Fig. 4 B **Nucleus**

β-actin 45kDa

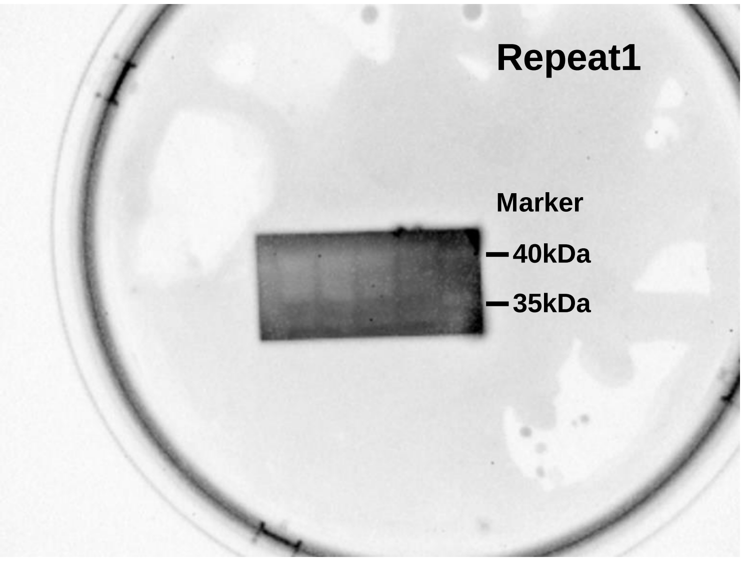

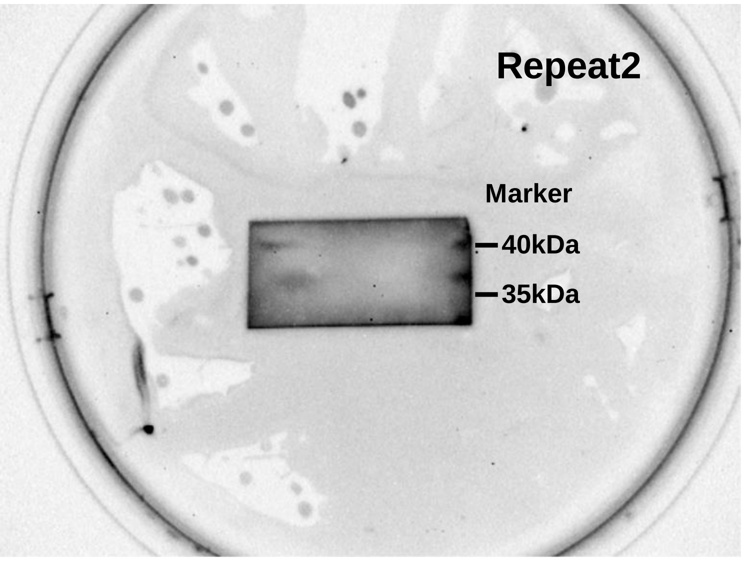

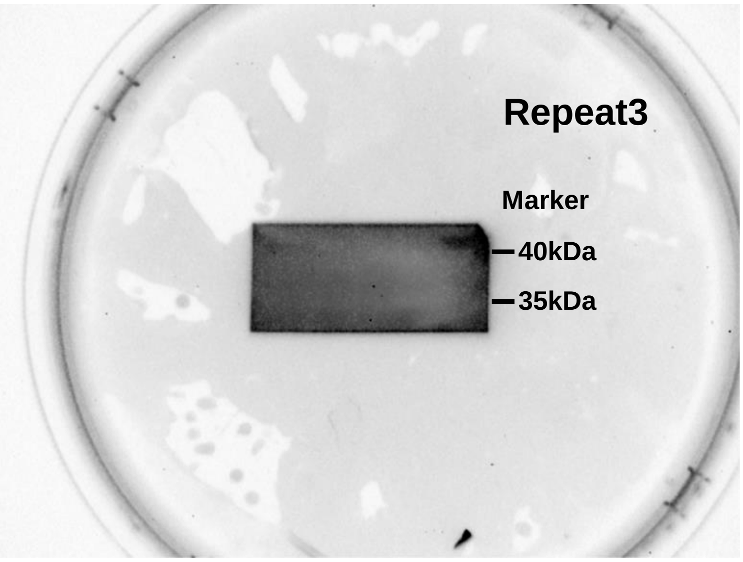

Fig. 5 B **Cytoplasm**

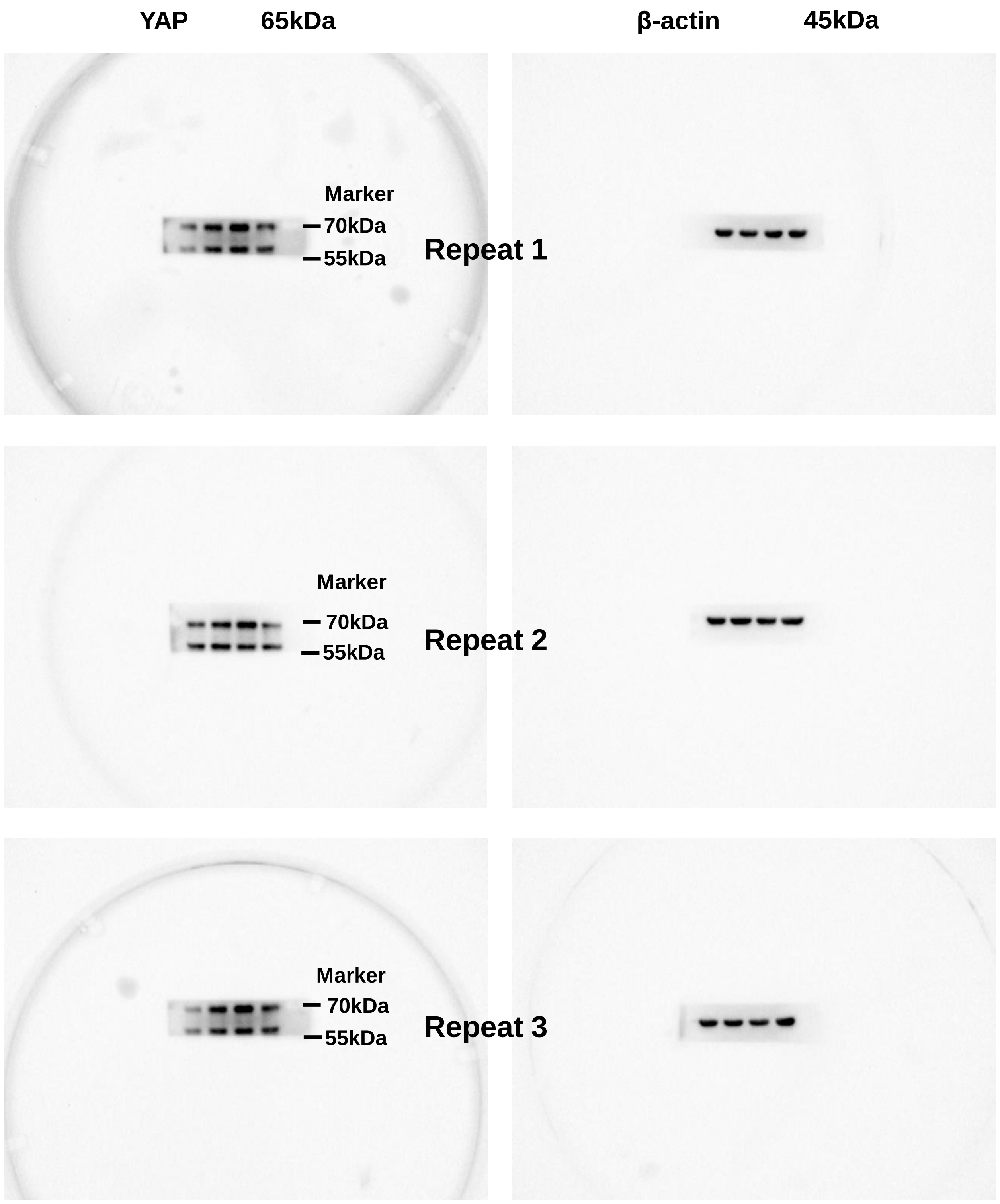

Fig. 5 B **Cytoplasm**

Histone H3 17kDa

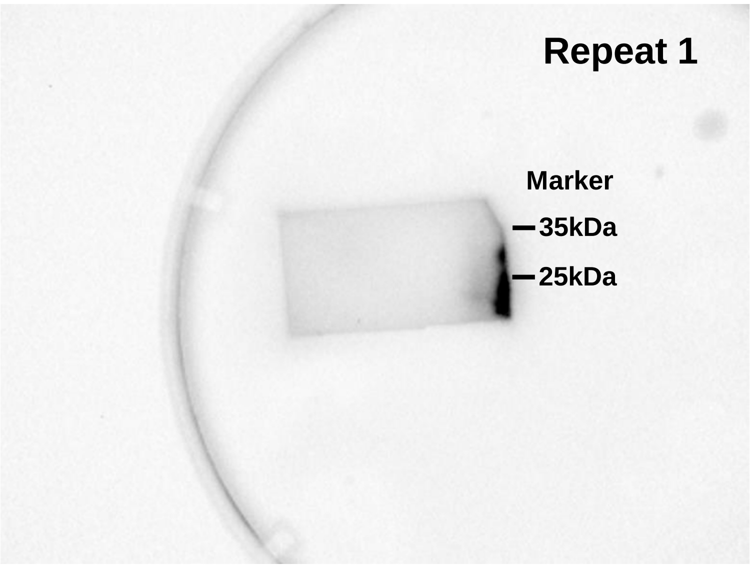

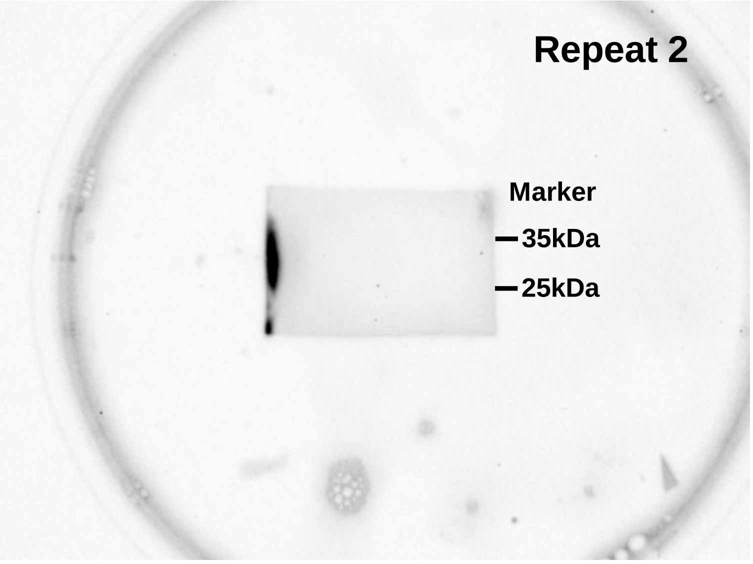

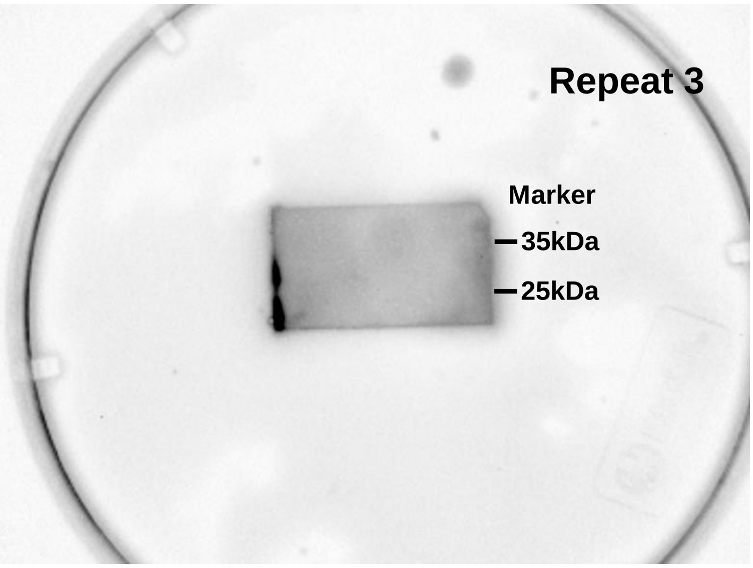

Fig. 5 B **Nucleus**

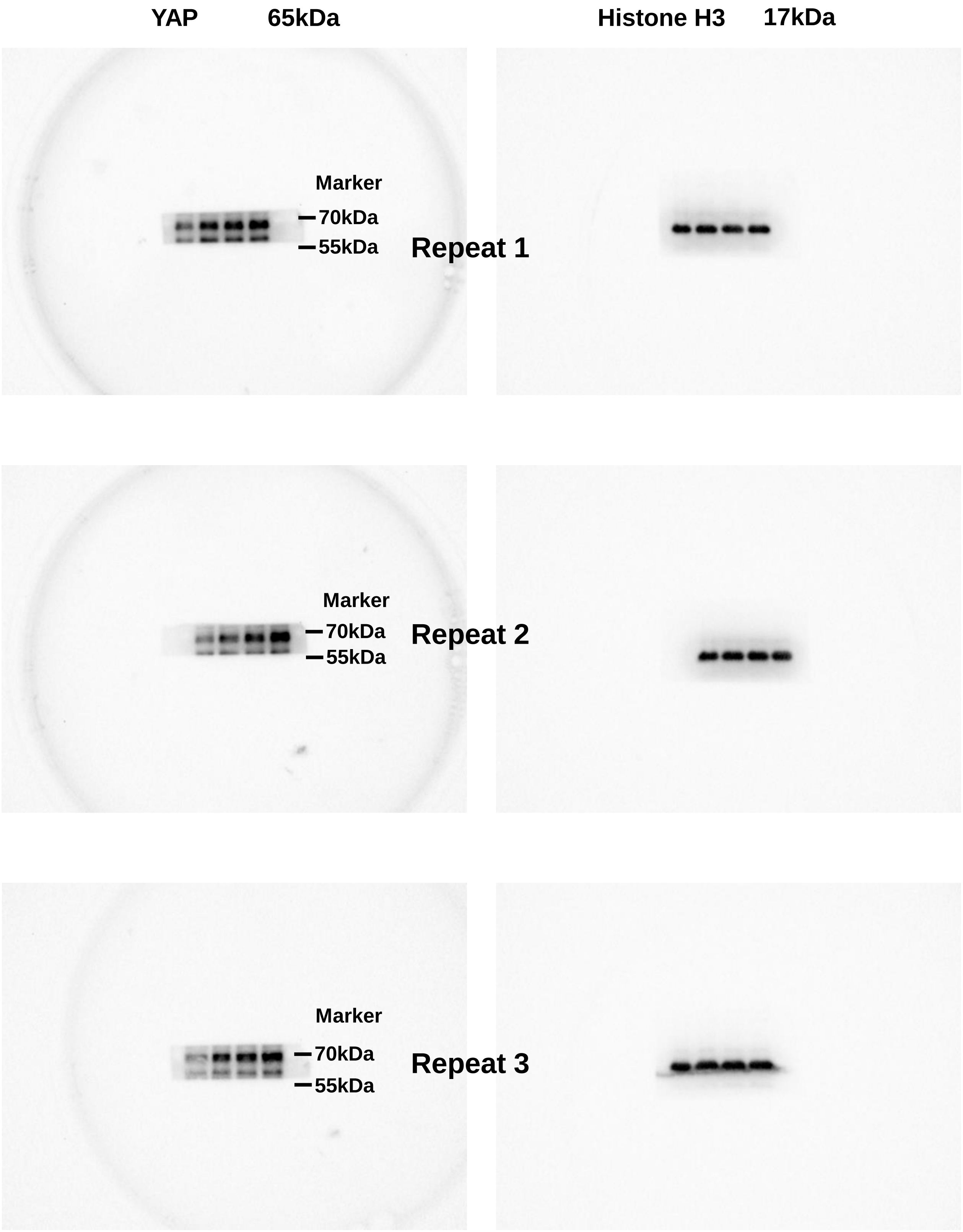

Fig. 5 B **Nucleus**

β-actin **45kDa**

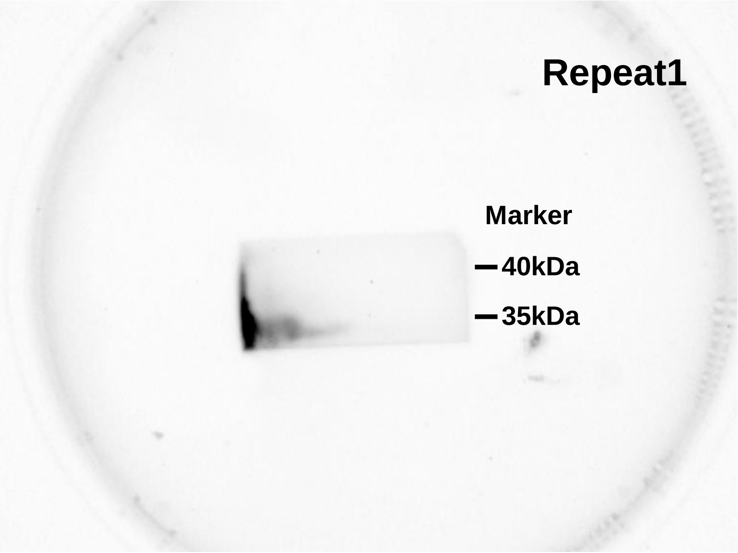

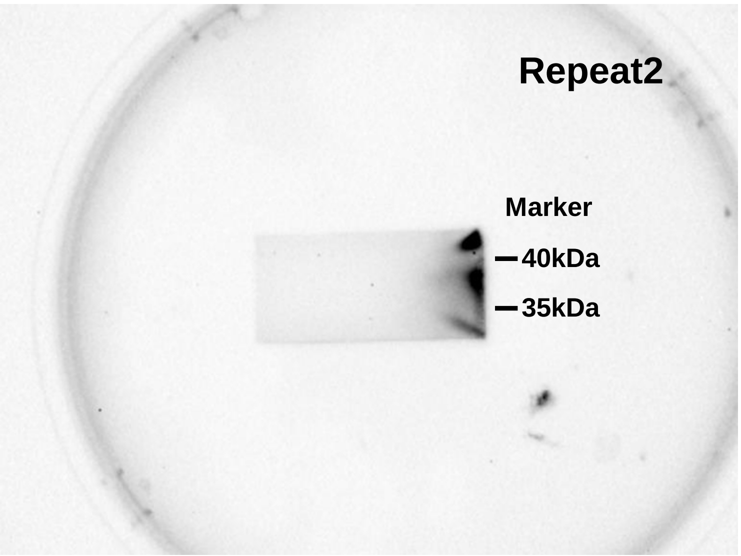

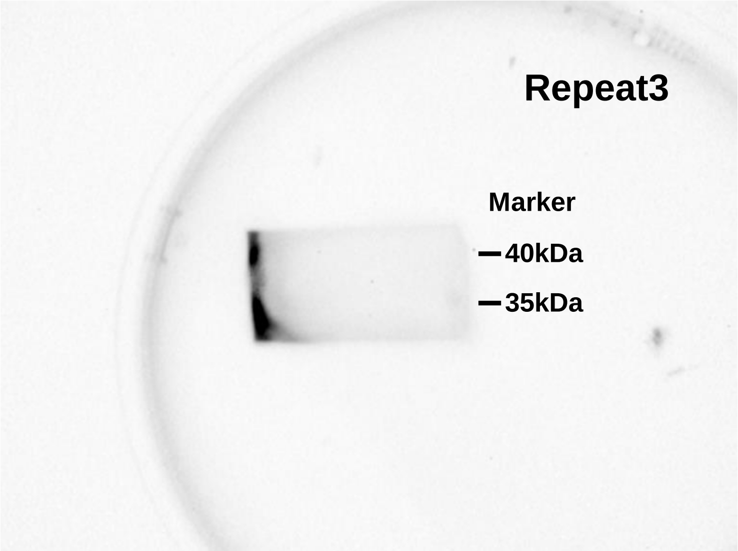

Fig. 6 A

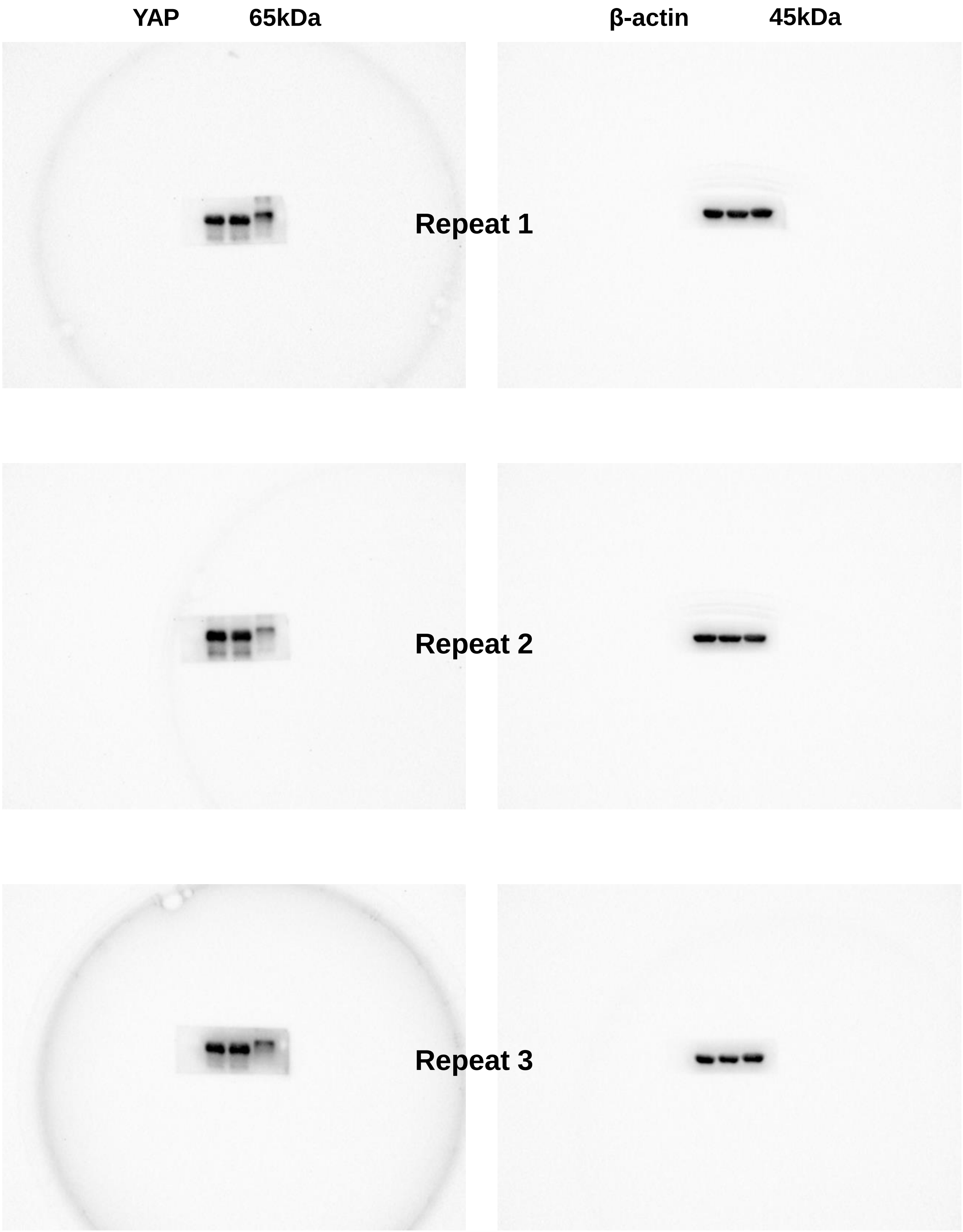

Fig. 6 B

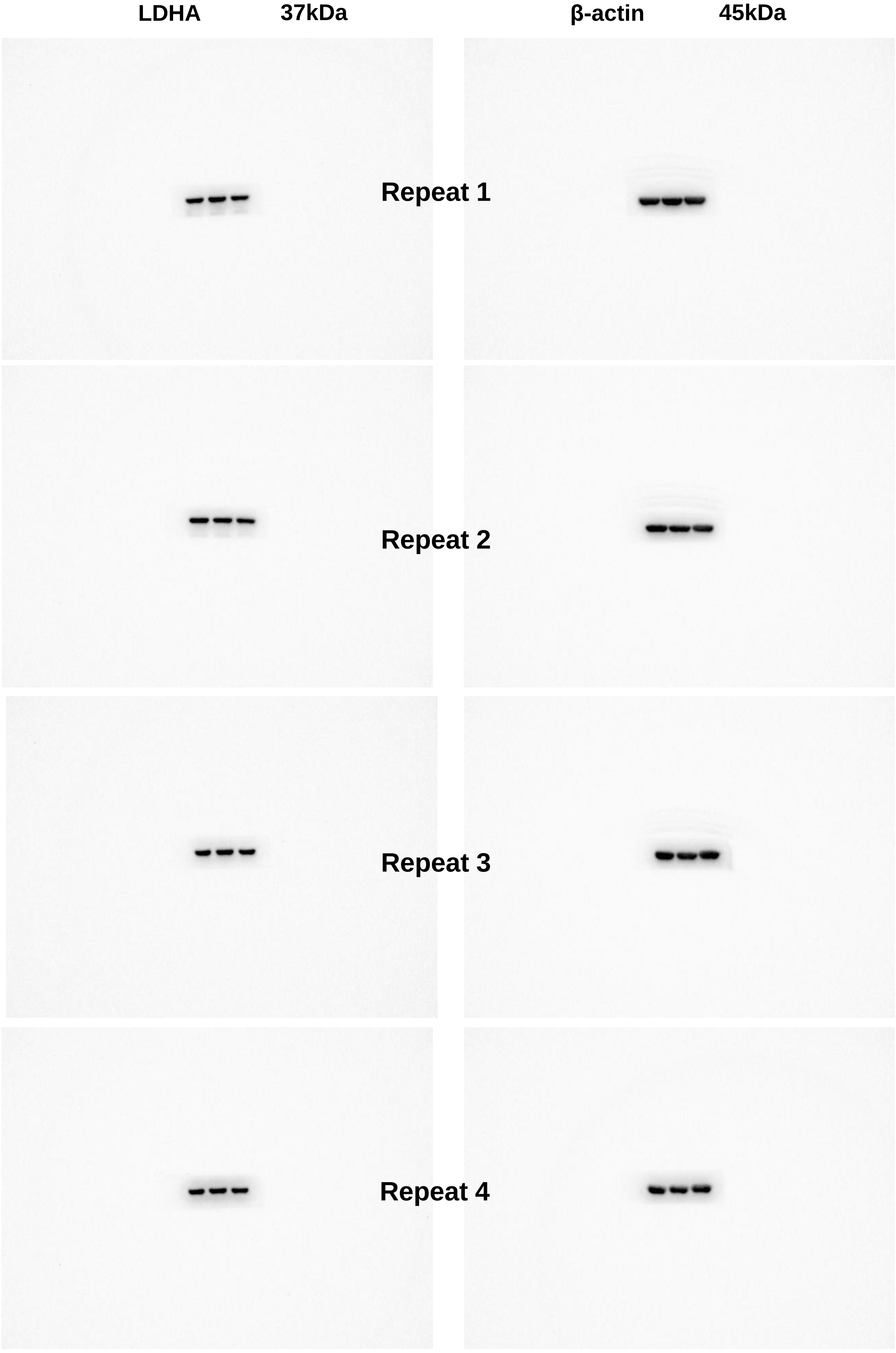

Fig. 6 C and D

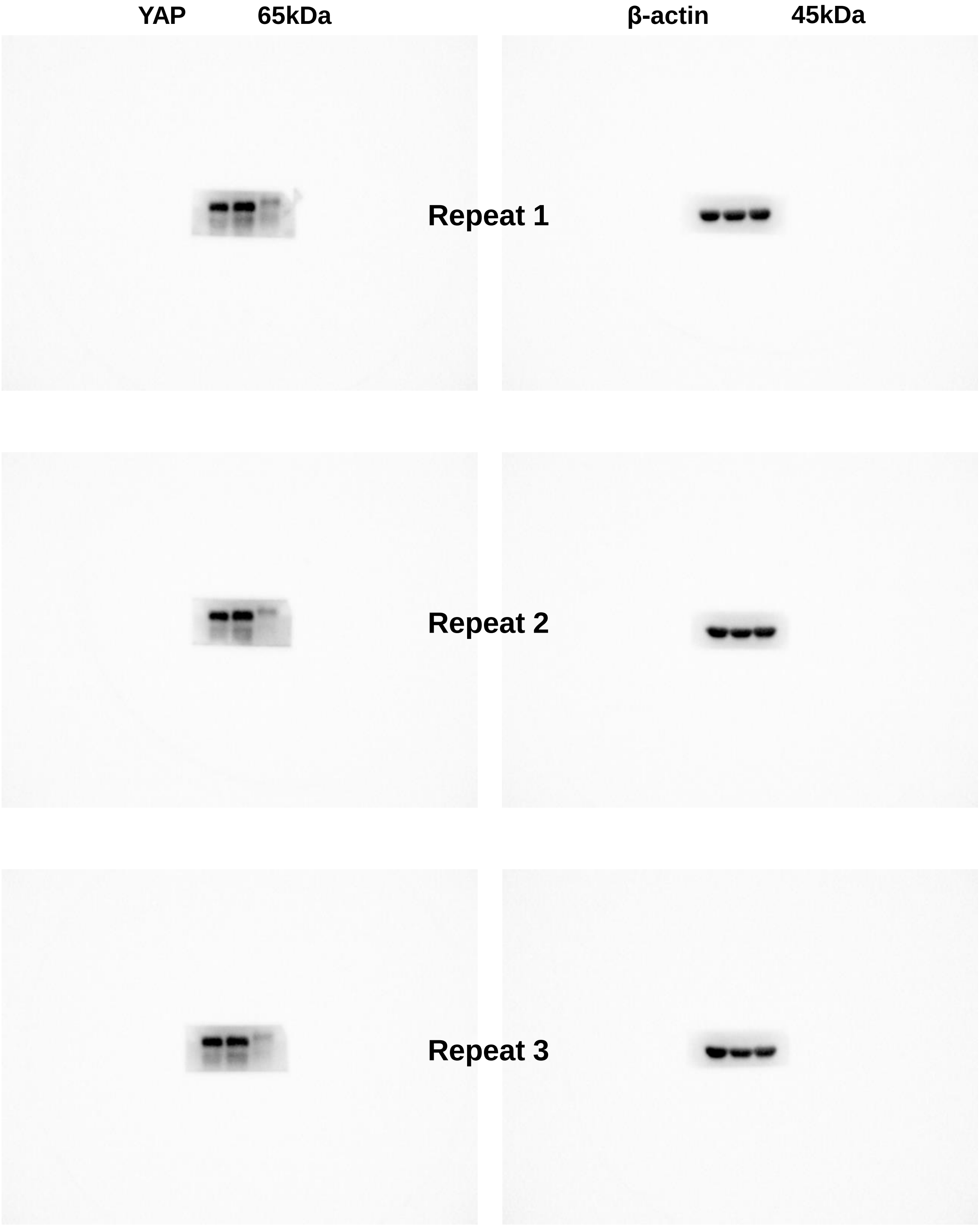

Fig. 6 C and E

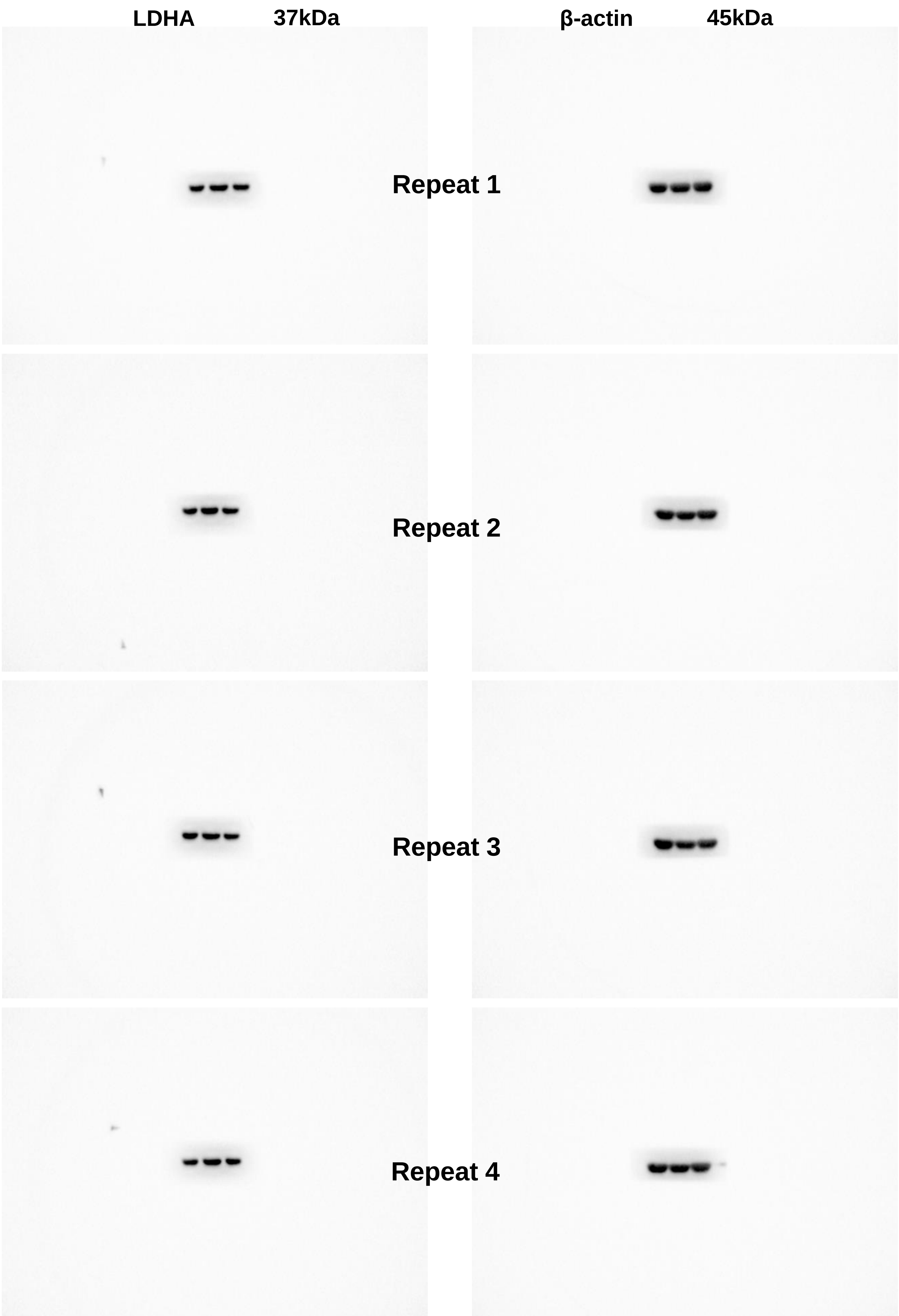

S1 Fig. A

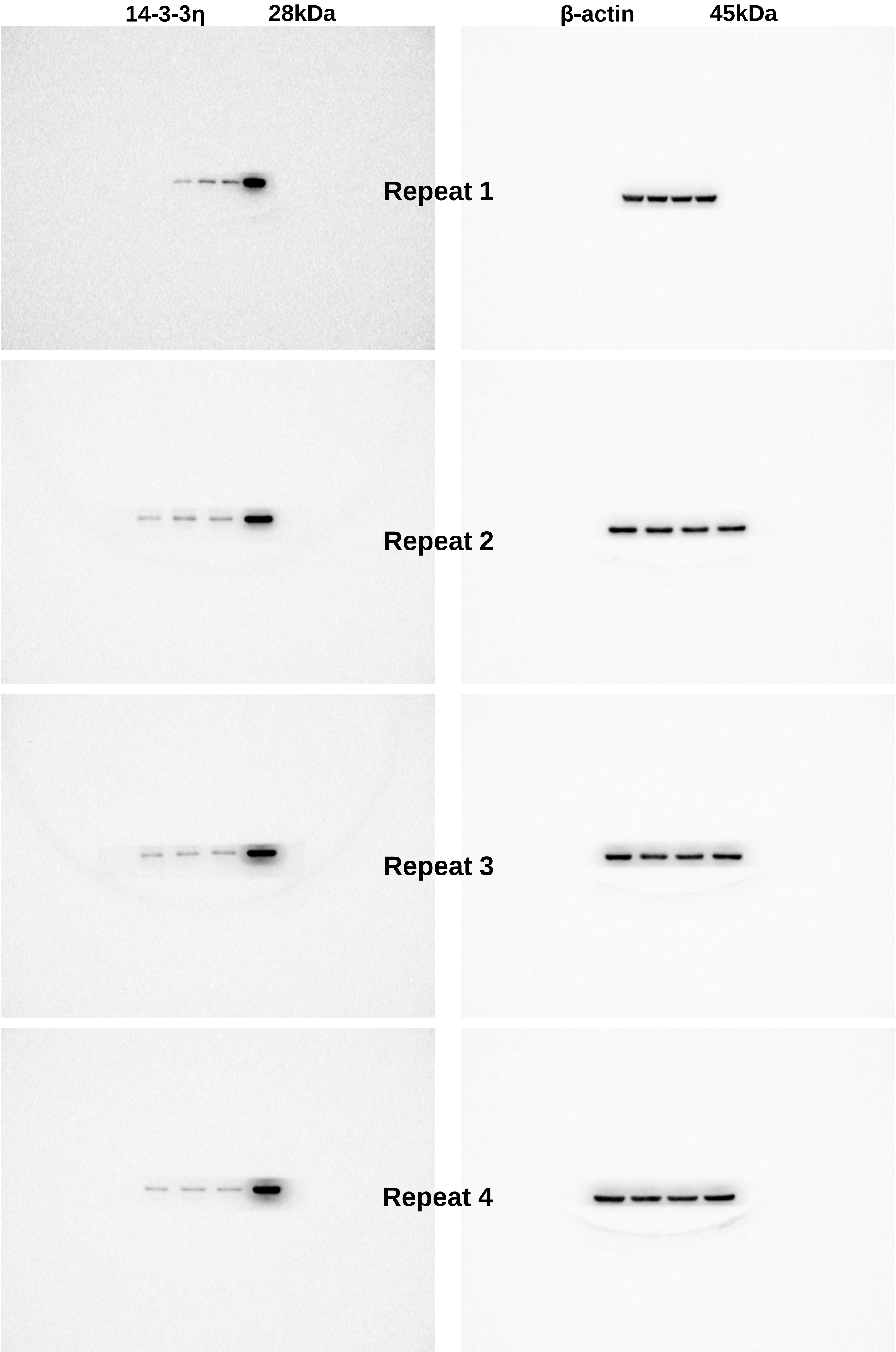

S1 Fig. D

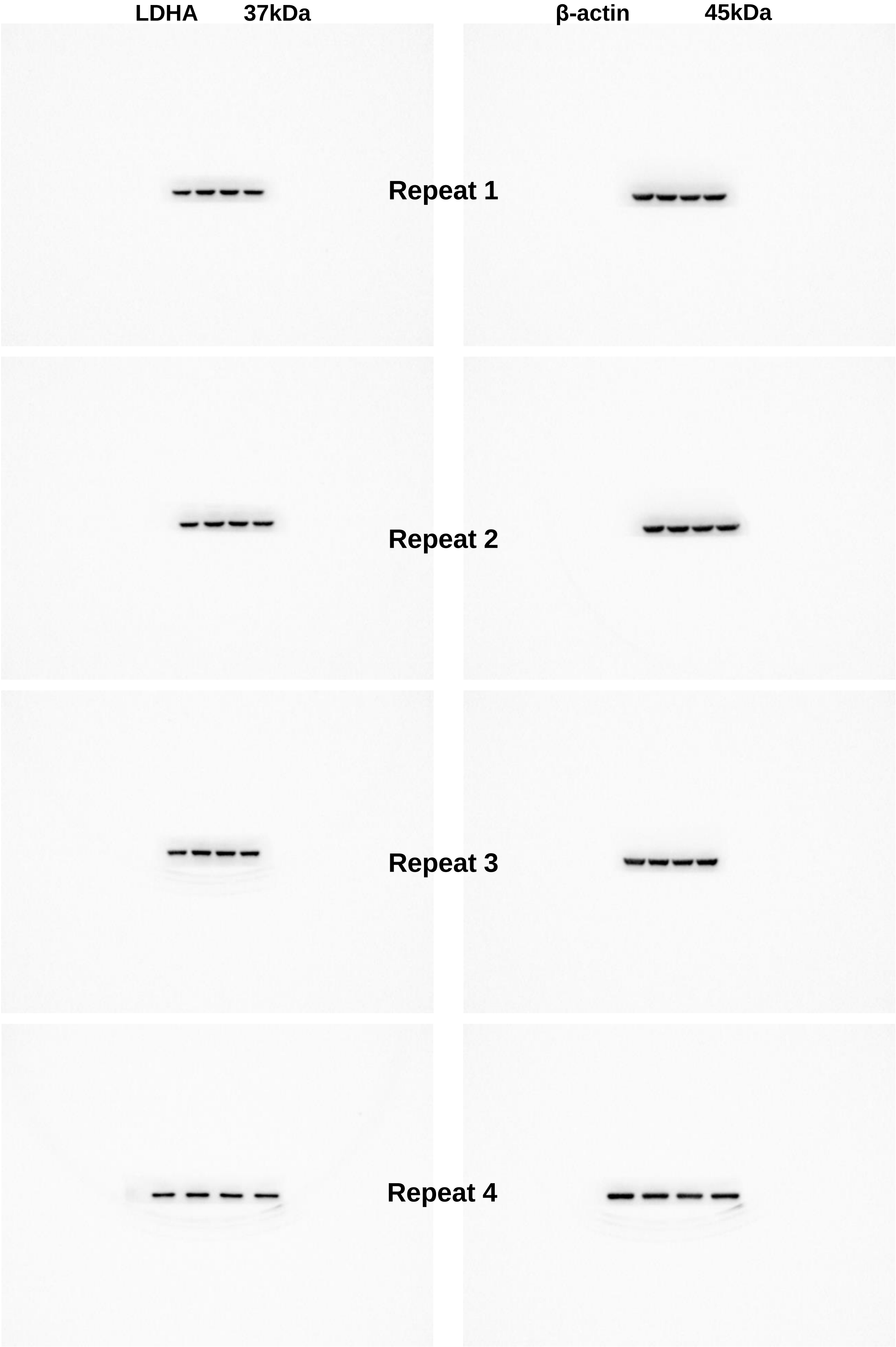

S1 Fig. F

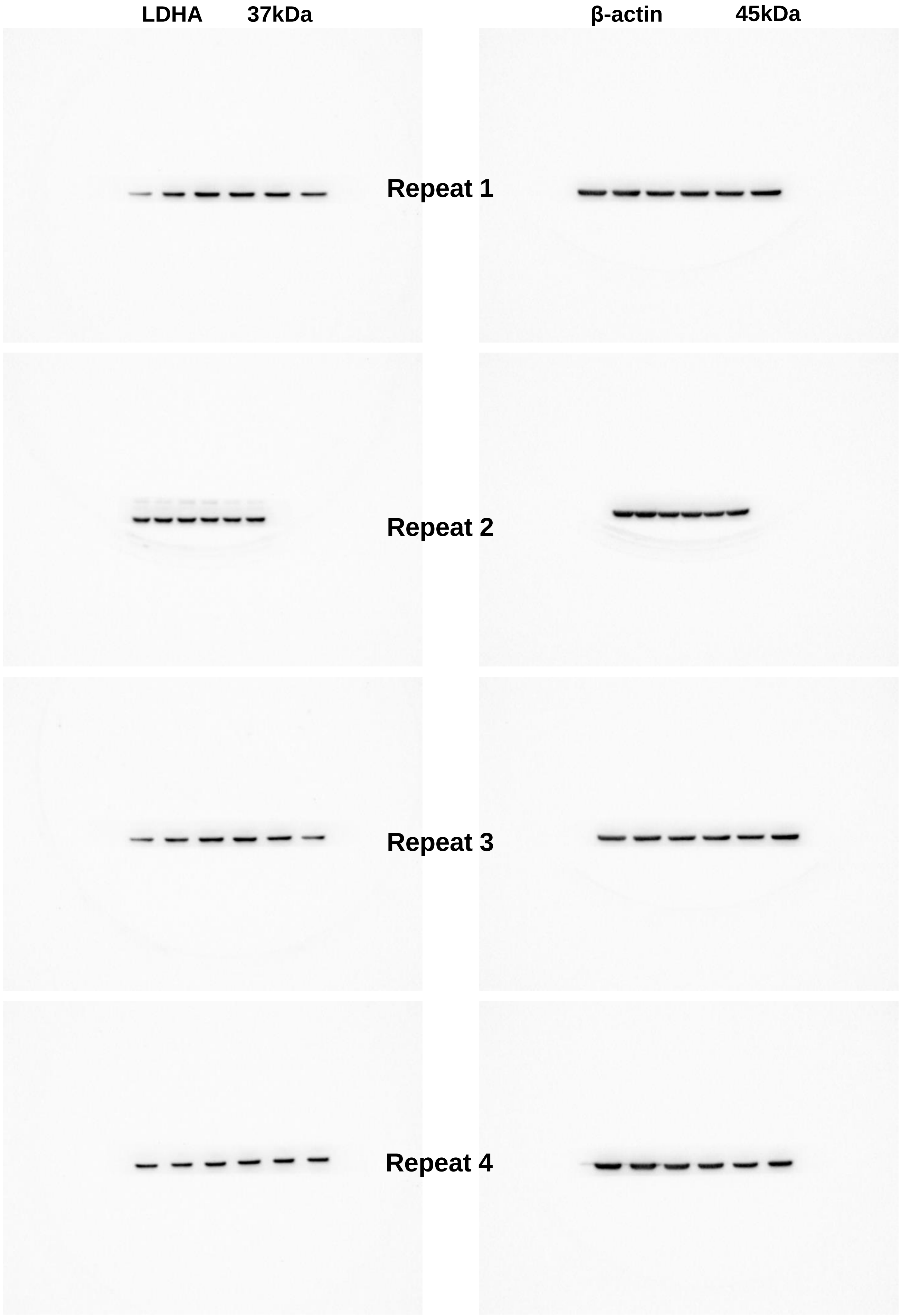

S4 Fig. A

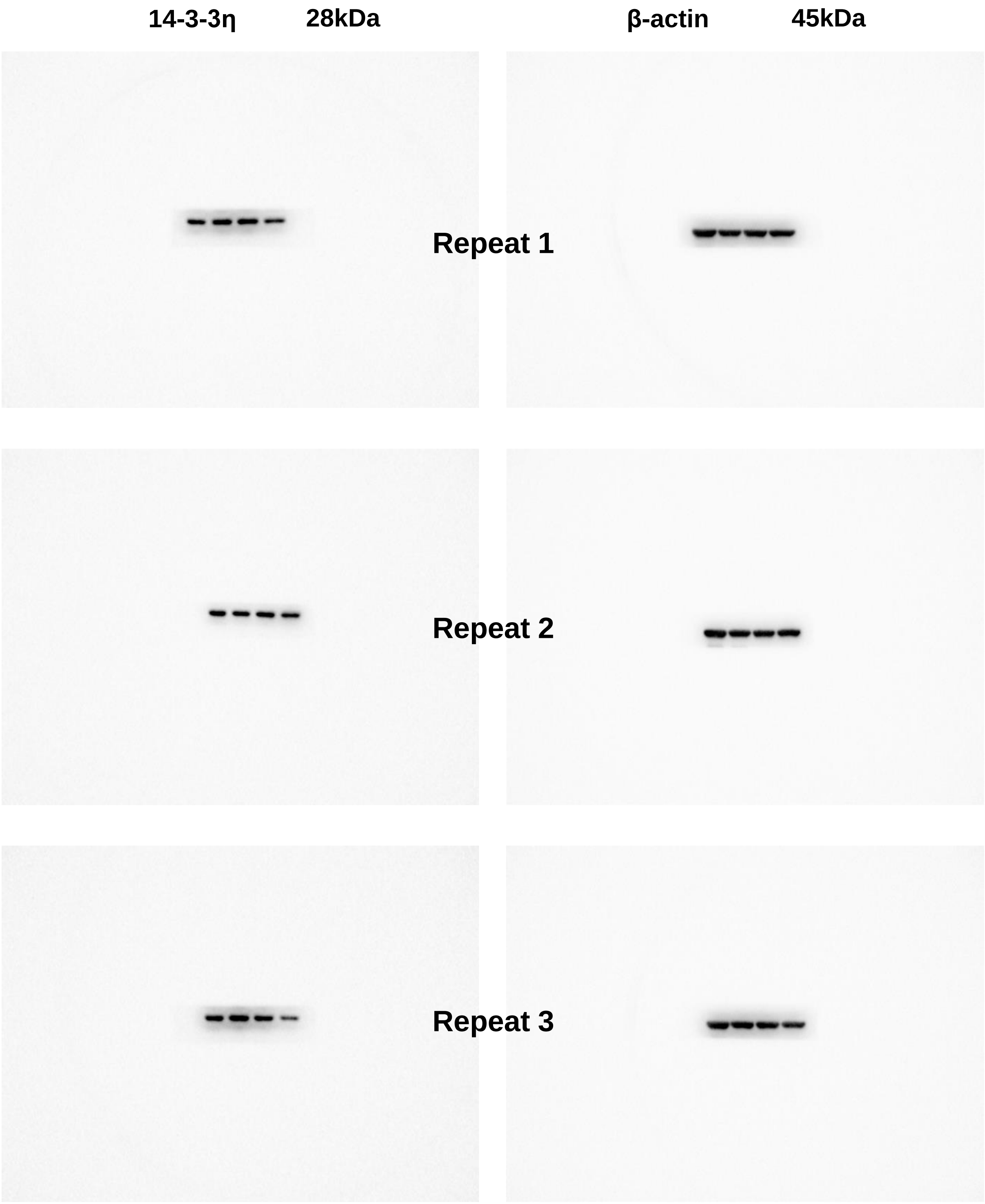

S4 Fig. A

14-3-3η **28kDa** **β-actin** **45kDa**

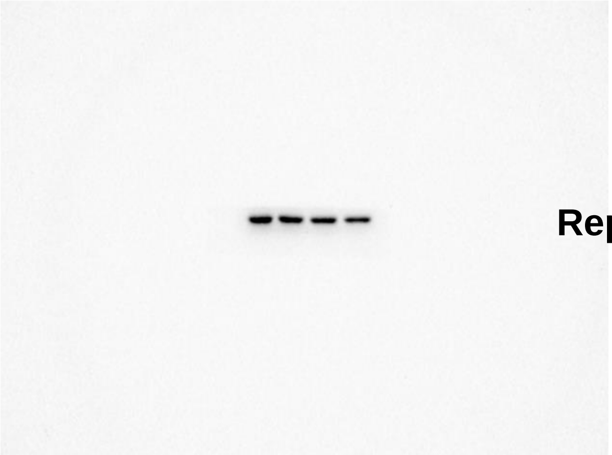

Repeat 4

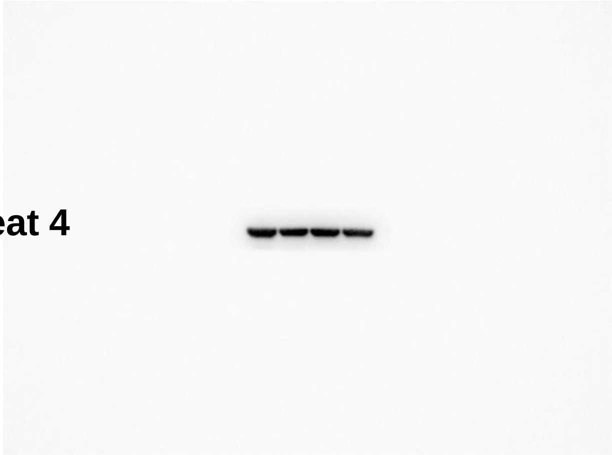

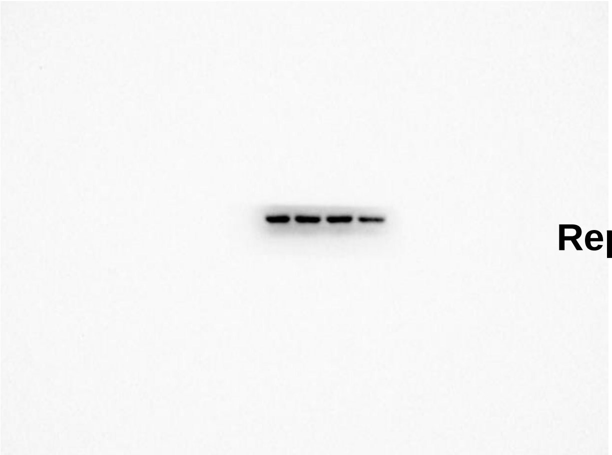

Repeat 5

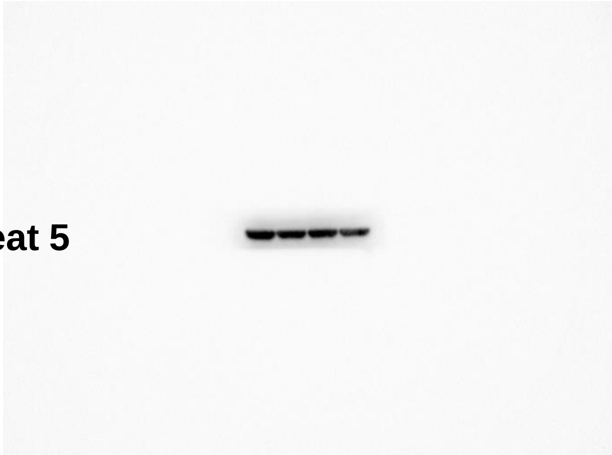

S4 Fig. D

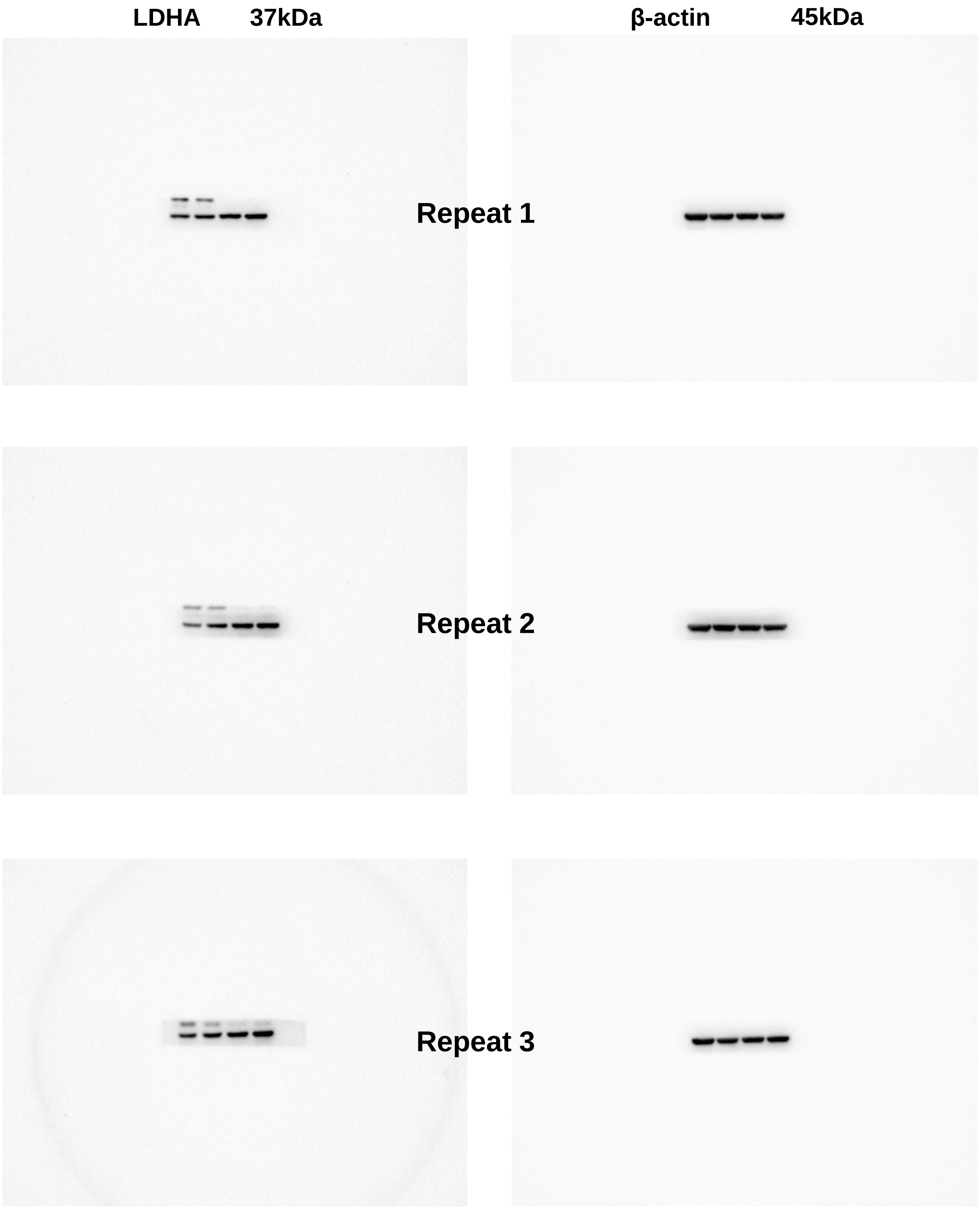

S4 Fig. F

LDHA 37kDa β-actin 45kDa

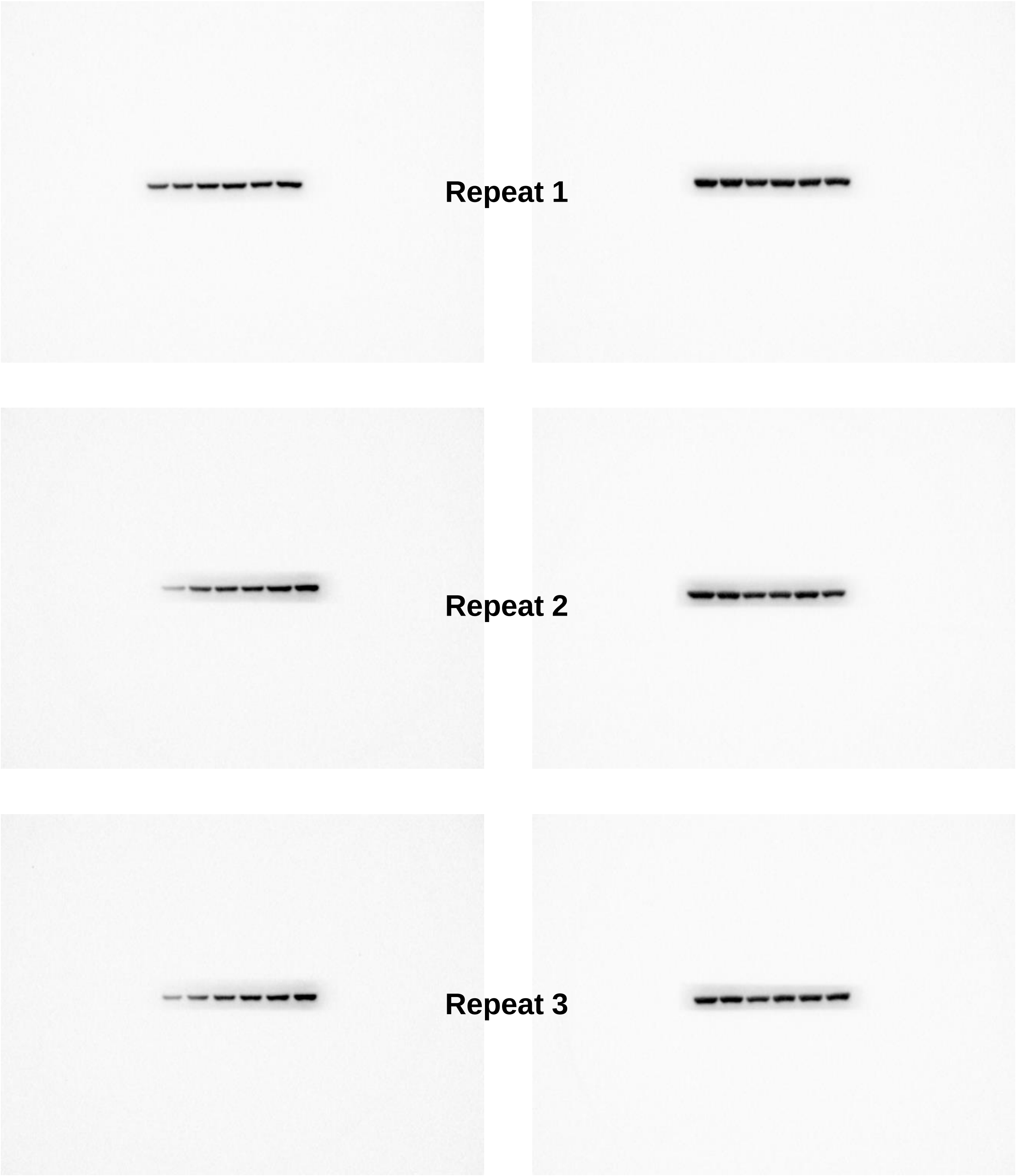

S4 Fig. F

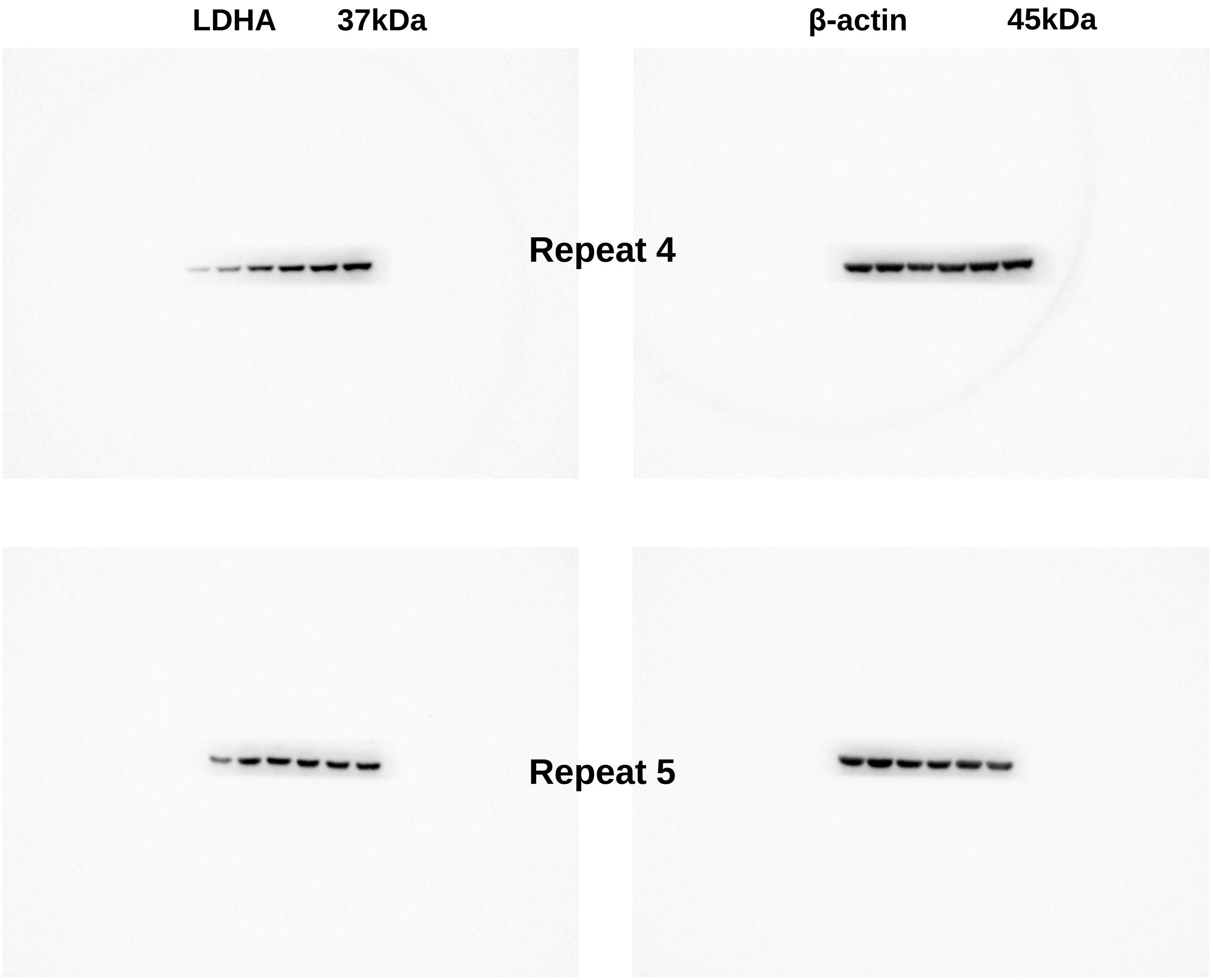

Supplement: S1 Raw image — (PDF) [file pone.0307696.s008.pdf]
